# Selected ‘Starter kit’ energy system modelling data for selected countries in Africa, East Asia, and South America (#CCG, 2021)

**DOI:** 10.1016/j.dib.2022.108021

**Published:** 2022-03-09

**Authors:** Lucy Allington, Carla Cannone, Ioannis Pappis, Karla Cervantes Barron, Will Usher, Steve Pye, Edward Brown, Mark Howells, Miriam Zachau Walker, Aniq Ahsan, Flora Charbonnier, Claire Halloran, Stephanie Hirmer, Jennifer Cronin, Constantinos Taliotis, Caroline Sundin, Vignesh Sridharan, Eunice Ramos, Maarten Brinkerink, Paul Deane, Andrii Gritsevskyi, Gustavo Moura, Arnaud Rouget, David Wogan, Edito Barcelona, Taco Niet, Holger Rogner, Franziska Bock, Jairo Quirós-Tortós, Jam Angulo-Paniagua, Satheesh Krishnamurthy, John Harrison, Long Seng To

**Affiliations:** a STEER Centre, Department of Geography & Environment, Loughborough University, UK; b KTH Royal Institute of Technology, Sweden; c University of Cambridge, UK; d University College London, UK; e Imperial College London, UK; f University of Oxford, UK; g The Cyprus Institute, Cyprus; h University College Cork, Ireland; i International Atomic Energy Agency, Austria; j Federal University of Ouro Preto, Brazil; k International Energy Agency, France; l Asia Pacific Energy Research Centre, Japan; m School of Sustainable Energy Engineering, Simon Fraser University, Canada; n Deutsche Gesellschaft für Internationale Zusammenarbeit (GIZ) GmbH, Germany; o School of Electrical Engineering, University of Costa Rica, San José, Costa Rica; p School of Engineering & Innovation, The Open University, UK

**Keywords:** U4RIA, Renewable energy, Cost-optimization, Energy policy, OSeMOSYS

## Abstract

Energy system modeling can be used to develop internally-consistent quantified scenarios. These provide key insights needed to mobilise finance, understand market development, infrastructure deployment and the associated role of institutions, and generally support improved policymaking. However, access to data is often a barrier to starting energy system modeling, especially in developing countries, thereby causing delays to decision making. Therefore, this article provides data that can be used to create a simple zero-order energy system model for a range of developing countries in Africa, East Asia, and South America, which can act as a starting point for further model development and scenario analysis. The data are collected entirely from publicly available and accessible sources, including the websites and databases of international organisations, journal articles, and existing modeling studies. This means that the datasets can be easily updated based on the latest available information or more detailed and accurate local data. As an example, these data were also used to calibrate a simple energy system model for Kenya using the Open Source Energy Modeling System (OSeMOSYS) and three stylized scenarios (Fossil Future, Least Cost and Net Zero by 2050) for 2020–2050. The assumptions used and the results of these scenarios are presented in the appendix as an illustrative example of what can be done with these data. This simple model can be adapted and further developed by in-country analysts and academics, providing a platform for future work.

**Specifications Table**

**Table:** 

Subject	Energy
Specific subject area	Energy System Modelling
Type of data	Tables Graphs Charts Description of main modelling assumptions
How data were acquired	Literature survey (databases and reports from international organisations; journal articles)
Data format	Raw and Analysed
Description of data collection	Data were collected from websites, annual reports and databases of international organisations, as well as academic articles and existing modelling databases. Open and accessible data sources were preferred. Data were collected and manipulated based on the inputs required to build an OSeMOSYS energy system model as described in a separate article referenced in Section 1. However, the data available through this document is independent of the tools and models. Units were checked to be consistent across all entries.
Data source location	Raw data sources are listed in Table 1 of this article.
Data accessibility	With the article and in a repository. Repository name: Zenodo Data identification number: 10.5281/zenodo.5820134 Direct URL to data: https://doi.org/10.5281/zenodo.5820133

**Value of the Data**

• Can be used to develop national energy system models to inform national energy investment outlooks and policy plans and provide insights on the evolution of the electricity supply system under different trajectories.
• Useful for country energy system analysts, policymakers and the broader scientific community as a zero-order starting point for model development.
• Can be used to examine a range of possible energy system pathways, in addition to the case studies given in this study, to provide a further understanding of the evolution of the country's power system.
• Useful for analysing the power system but also for capacity building activities. The methodology of translating the input data into modeling assumptions for a cost-optimization tool is presented in the appendix, which helps develop a zero-order Tier 2 national energy model [1] (source A) consistent with U4RIA energy planning goals [2].
• Useful for accelerating teaching activities, consultations, and government policy analysis in the energy planning field as evidenced by research that has been based on these data, including assessment of wind power in Morocco [3], assessment of NDC targets in Ghana [4], and assessment of decarbonisation pathways in Kenya [5].
• By combining secondary data from multiple, diverse sources, the work provides analysts with complete and accessible datasets, helping to overcome barriers of data inaccessibility.

## 1 Data Description

The data provided can be used as input data to develop an energy system model for the included countries in Africa, South America, and Asia. These countries are selected based on geography and data availability. This paper presents selected country-specific data and related aggregated data by region, with an example energy system model in the appendix; however, additional more comprehensive country-specific datasets are available externally for each country (see Appendix B for links to each available country-specific dataset, which should be consulted by those wishing to use these data for their own country analyses). As an illustration, these data were used to develop an example energy system model for Kenya using the cost-optimization tool OSeMOSYS [6] for 2015–2050. For reference, that model is described in Appendix A, and its data files are available as supplementary materials. The data provided were collected from publicly available sources, including the reports of international organizations, journal articles and existing model databases. The methods of data collection and preparation are described in Section 2 of this article and a separate article that provides guidance to those wishing to create similar datasets for other countries [7]. The data sources used are listed in Table 1; each data source is assigned a letter code which is then referred to in the text. The dataset includes the techno-economic parameters of supply-side technologies, installed capacities, emissions factors and final electricity demands.

**Table 1:** Data sources used in this article. In the text, lettered data sources corresponding to those in Table 1 are included in brackets.

Source ID	Reference
A	C. Cannone, Towards evidence-based policymaking: energy modeling tools for sustainable development, UPC Barcelona (2020). http://hdl.handle.net/2117/333306
B	M. Brinkerink, P. Deane, PLEXOS-World 2015. (2020). https://doi.org/10.7910/DVN/CBYXBY
C	M. Brinkerink, B. Gallachóir, P. Deane, Building and Calibrating a Country-Level Detailed Global Electricity Model Based on Public Data, Energy Strateg Rev. 33 (2021) 100592. https://doi.org/10.1016/j.esr.2020.100592
D	L. Byers, J. Friedrich, R. Hennig, A. Kressig, X. Li, C. McCormick, et al., A Global Database of Power Plants, Washington, DC (2018). https://www.wri.org/publication/global-power-plant-database
E	IRENA, Renewable Energy Statistics 2020, Abu Dhabi (2020). https://www.irena.org/publications/2020/Jul/Renewable-energy-statistics-2020
F	IRENA, Planning and Prospects for Renewable Power in Eastern and Southern Africa, Abu Dhabi (2021). https://www.irena.org/-/media/Files/IRENA/Agency/Publication/2021/Apr/IRENA_Planning_Prospects_Africa_2021.pdf
G	IRENA, Planning and prospects for renewable power: West Africa, Abu Dhabi (2018). https://www.irena.org/-/media/Files/IRENA/Agency/Publication/2018/Nov/IRENA_Planning_West_Africa_2018.pdf
H	IRENA, Future of Wind, Abu Dhabi (2019). https://www.irena.org/-/media/Files/IRENA/Agency/Publication/2019/Oct/IRENA_Future_of_wind_2019.pdf
I	IRENA, ASEAN Centre for Energy, Renewable Energy Outlook for ASEAN, Abu Dhabi (2016). https://www.irena.org/-/media/Files/IRENA/Agency/Publication/2016/IRENA_REmap_ASEAN_2016_report.pdf
J	IRENA, Renewable Power Generation Costs in 2019, Abu Dhabi (2020). https://irena.org/-/media/Files/IRENA/Agency/Publication/2020/Jun/IRENA_Power_Generation_Costs_2019.pdf
K	G. N. P. de Moura, L.F.L. Legey, M. Howells, A Brazilian perspective of power systems integration using OSeMOSYS SAMBA – South America Model Base – and the bargaining power of neighbouring countries: A cooperative games approach, Energy Policy. 115 (2018) 470–485. https://doi.org/10.1016/j.enpol.2018.01.045
L	I. Staffell, S. Pfenninger, Using bias-corrected reanalysis to simulate current and future wind power output, Energy. 114 (2016) 1224–1239. https://doi.org/10.1016/j.energy.2016.08.068
M	I. Staffell, S. Pfenninger, Long-term patterns of European PV output using 30 years of validated hourly reanalysis and satellite data. Energy. 114 (2016). 1251–1265. https://doi.org/10.1016/j.energy.2016.08.060
N	I. Pappis, V. Sridharan, W. Usher, M. Howells, KTH-dESA/jrc_temba: TEMBA 2.0 (Version v2.0.3). (2021). https://github.com/KTH-dESA/jrc_temba/releases/tag/v2.0.3
O	National Renewable Energy Laboratory, Global CFDDA-based Onshore and Offshore Wind Potential Supply Curves by Country, Class, and Depth (quantities in GW and PWh). (2014). https://openei.org/doe-opendata/dataset/c186913f-6684-4455-a2f2-f26e152a9b35/resource/4dc4a6fd-3a63-47df-bcbe-e9c83b83b38e/download/nrelcfddawindsc20130603.xlsx
P	IRENA, Future of wind: Deployment, investment, technology, grid integration and socio-economic aspects (A Global Energy Transformation paper), Abu Dhabi.(2019).https://www.irena.org/-/media/Files/IRENA/Agency/Publication/2019/Oct/IRENA_Future_of_wind_2019.pdf
Q	I. Pappis, M. Howells, V. Sridharan, F. Gardumi, E. Ramos, W. Usher, et al., Energy projections for African countries. (2019). https://publications.jrc.ec.europa.eu/repository/handle/JRC118432
R	International Energy Agency, IndexMundi, Electric Power Transmission and Distribution Losses (% of output) - Country Ranking – Asia. (2018). https://www.indexmundi.com/facts/indicators/EG.ELC.LOSS.ZS/rankings/asia
S	Y. Li, Y. Chang Y, Infrastructure Investments for Power Trade and Transmission in ASEAN+2: Costs, Benefits, Long-Term Contracts, and Prioritised Development. (2014). https://www.eria.org/ERIA-DP-2014-21.pdf
T	McKinsey, McKinsey Refinery Reference Desk. (2020). [Accessed 13/03/2021]. https://www.mckinseyenergyinsights.com/resources/refinery-reference-desk/
U	IEA ETSAP, Oil Refineries. (2014). https://iea-etsap.org/E-TechDS/PDF/P04_Oil Ref_KV_Apr2014_GSOK.pdf
V	U.S. EIA, Assumptions to the Annual Energy Outlook 2020: International Energy Module.(2020). https://www.eia.gov/outlooks/aeo/assumptions/pdf/international.pdf
W	Asia-Pacific Economic Cooperation, APEC Energy Demand and Supply Outlook 7th Edition. (2019). https://aperc.or.jp/publications/reports/outlook.php
X	ERIA, Cost Analysis of Biomass Power Generation. (2019). https://www.eria.org/uploads/media/12_RPR_FY2018_09_Chapter_5.pdf
Y	Argus, Argus Biomass Markets Weekly Biomass Market News and Analysis Issue 20-47. (2020). https://www.argusmedia.com/-/media/Files/sample-reports/argus-biomassmarkets.ashx?la=en&hash=872E2C03A0A78FE3F236BBF00E7729E3114326E0
Z	P. Howes, J. Bates, A. Brown, R. Diaz-Chavez, S. Christie, A. Bayley, Global Biomass Markets Final Report. (2018). https://assets.publishing.service.gov.uk/government/uploads/system/uploads/attachment_data/file/795029/Global_Biomass_Markets_Final_report.pdf
AA	IPCC, Emission Factor Database. [accessed 03/02/2021]. https://www.ipcc-nggip.iges.or.jp/EFDB/main.php
AB	S. Hermann, A. Miketa, N. Fichaux, Estimating the Renewable Energy Potential in Africa, Abu Dhabi. (2014). https://www.irena.org/-/media/Files/IRENA/Agency/Publication/2014/IRENA_Africa_Resource_Potential_Aug2014.pdf
AC	IRENA, Analysis of Infrastructure for Renewable Power in Eastern and Southern Africa, Abu Dhabi. (2015). https://www.irena.org/-/media/Files/IRENA/Agency/Publication/2015/IRENA_Africa_CEC_infrastructure_2015.pdf
AD	United Nations, World Small Hydropower Development Report 2019. (2019). https://www.unido.org/our-focus-safeguarding-environment-clean-energy-access-productive-use-renewable-energy-focus-areas-small-hydro-power/world-small-hydropower-development-report
AE	V. Veng, B. Suryadi, A. Damar Pranadi, A review of renewable energy development and its policy under nationally determined contributions in ASEAN, Int J Smart Grids Clean Energy. (2019). https://accept.aseanenergy.org/wp-content/uploads/2020/01/A-Review-of-RE-and-NDCs-in-ASEAN.pdf
AF	NREL, Exploring Renewable Energy Opportunities in Select Southeast Asian Countries. (2019). https://www.osti.gov/biblio/1527336-exploring-renewable-energy-opportunities-select-southeast-asian-countries-geospatial-analysis-levelized-cost-energy-utility-scale-wind-solar-photovoltaics
AG	NREL, Solar Resources by Class and Country. (2014). https://openei.org/datasets/dataset/solar-resources-by-class-and-country
AH	The World Bank, energydata.info. (2019) [accessed 03/02/2021]. https://energydata.info/en
AI	US EIA, US Energy Information Administration. (2019). [accessed 13/3/2021]. https://www.eia.gov/
AJ	Worldometer, Worldometer. (2020). [accessed 13/03/2021]. https://www.worldometers.info/
AK	BP, Full report – BP Statistical Review of World Energy 2019. (2019). https://www.bp.com/content/dam/bp/business-sites/en/global/corporate/pdfs/energy-economics/statistical-review/bp-stats-review-2019-full-report.pdf
AL	International Energy Agency, IEA Sankey Diagram. (2019). [accessed 13/03/2021]. https://www.iea.org/sankey/
AM	OLADE, Energy Outlook of Latin America and the Caribbean 2019. (2019). http://biblioteca.olade.org/opac-tmpl/Documentos/old0446b.pdf
AN	A. Shivakumar, M. Brinkerink, T. Niet, W. Usher, OSeMOSYS/osemosys_global: Development release for CCG (Version v0.2.b0). (2021). https://zenodo.org/record/4624417#.Yd2pQmjP02w
AO	IRENA, Southern African Power Pool: Planning and Prospects for Renewable Power, Abu Dhabi. (2013). https://www.irena.org/-/media/Files/IRENA/Agency/Publication/2013/SAPP.pdf
AP	United Nations Development Programme Asia-Pacific Regional Centre, Achieving Sustainable Energy For All in the Asia-Pacific. (2013). https://www.asia-pacific.undp.org/content/rbap/en/home/library/climate-and-disaster-resilience/APRC-EE-2013-SE4ALL.html
AQ	Global Electrification Platform, Explore — Global Electrification Platform. (2019) [accessed 13/03/2021]. https://electrifynow.energydata.info/explore/ng-1?year=2030&scenario=0_0_0_0_0_0&filters=r8_2766837%7Cr0_190%7C1_3_5_6_7%7Cr0_131%7Cr0_68%7Cr15_2105
AR	NREL, Annual Technology Baseline 2020 Data. (2020). https://atb.nrel.gov/electricity/2020/data.php
AS	E. Terpilowski-Gill, Decarbonising the Laotian Energy System, Imperial College London. (2020). http://hdl.handle.net/10044/1/86671
AT	O. Okolo, H. Teng, Analysing Nigeria's Energy system in light of the UN's Sustainable Development Goals. (2017) https://www.diva-portal.org/smash/get/diva2:1131269/FULLTEXT01.pdf

U4RIA are practical goals designed to improve energy modeling for policy support through guidelines and best practices [2]. They are short for Ubuntu (meaning community focused), Retrievability, Reusability, Repeatability, Interoperability and Auditability. The datasets and example model move to meet U4RIA goals in that partially:

• We develop examples of results that can be used by other research communities, including energy and transport, and to aid mitigation strategies.
• The illustrative analyses are retrievable, reusable, repeatable.
• As data are defined, elements of interoperability are feasible.
• Moreover, the investigation could be audited or verified (not to say that it is ‘accurate’ but simply reproducible).

**Table:** 

Item	Description of Content

Table 1	A table showing the raw data sources that data were collected from
Table 2	A table showing the estimated installed capacity of different on-grid power plant types in selected countries in Africa in 2018
Table 3	A table showing the estimated installed capacity of different on-grid power plant types in selected countries in East Asia in 2018
Table 4	A table showing the estimated installed capacity of different on-grid power plant types in selected countries in South America in 2018
Table 5	A table showing the estimated installed capacity of off-grid solar PV and hydropower in selected countries in Africa in 2018
Table 6	A table showing the estimated installed capacity of off-grid solar PV and hydropower in selected countries in East Asia in 2018
Table 7	A table showing the estimated installed capacity of off-grid solar PV and hydropower in selected countries in East South America in 2018
Table 8	A table showing techno-economic parameters for electricity generation technologies in Africa
Table 9	A table showing techno-economic parameters for electricity generation technologies in South East Asia
Table 10	A table showing techno-economic parameters for electricity generation technologies in South America
Table 11	A table showing capital cost projections for renewable energy technologies in Africa up to 2050
Table 12	A table showing capital cost projections for renewable energy technologies in South East Asia up to 2050
Table 13	A table showing capital cost projections for renewable energy technologies in South America up to 2050
Table 14	A table showing estimated average capacity factors for solar PV, hydropower and wind in selected countries in Africa
Table 15	A table showing estimated average capacity factors for solar PV, hydropower and wind in selected countries in East Asia
Table 16	A table showing estimated average capacity factors for solar PV, hydropower and wind in selected countries in South America
Table 17	A table showing estimated combined efficiency of transmission and distribution in selected countries in Africa in 2020, 2030 & 2050
Table 18	A table showing estimated combined efficiency of transmission and distribution in selected countries in East Asia in 2020, 2030 & 2050
Table 19	A table showing estimated combined efficiency of transmission and distribution in selected countries in South America in 2020, 2030 & 2050
Table 20	A table showing estimated domestic refinery capacity for selected countries in Africa
Table 21	A table showing estimated domestic refinery capacity for selected countries in East Asia
Table 22	A table showing estimated domestic refinery capacity for selected countries in South America
Table 23	A table showing cost and performance data for refinery technologies
Table 24	A table showing fuel price projections in Africa up to 2050
Table 25	A table showing fuel price projections in East Asia up to 2050
Table 26	A table showing fuel price projections in South America up to 2050
Table 27	A table showing carbon dioxide emissions factors by fuel
Table 28	A table showing estimated renewable energy potentials for selected countries in Africa
Table 29	A table showing estimated renewable energy potentials for selected countries East in Asia
Table 30	A table showing estimated renewable energy potentials for selected countries in South America
Table 31	A table showing estimated fossil fuel reserves for selected countries in Africa
Table 32	A table showing estimated fossil fuel reserves for selected countries in Asia
Table 33	A table showing estimated fossil fuel reserves for selected countries in South Africa
Fig. 1	A graph showing a final electricity demand projection for selected countries in the North Africa Power Pool from 2015 to 2050
Fig. 2	A graph showing a final electricity demand projection for selected countries in the Central Africa Power Pool from 2015 to2050
Fig. 3	A graph showing a final electricity demand projection for selected countries in the East Africa Power Pool from 2015 to 2050
Fig. 4	A graph showing a final electricity demand projection for selected countries in the West Africa Power Pool from 2015 to 2050
Fig. 5	A graph showing a final electricity demand projection for selected countries in the South Africa Power Pool from 2015 to 2050
Fig. 6	A graph showing a final electricity demand projection for selected countries in East Asia from 2015 to 2050
Fig. 7	A graph showing a final electricity demand projection for selected countries in South America from 2015 to 2050

### 1.1 Existing electricity supply system

Various technologies can be used to generate electricity, with some using fuels such as oil or natural gas, and others making use of renewable energy sources, such as hydropower. These electricity generation technologies can either be on-grid technologies, which are generally larger in capacity and supply electricity to the national transmission grid to be transported to consumers, or off-grid technologies, which usually provide electricity directly to the consumer at the site of demand, for example roof-top solar PV panels. The estimated existing electricity generation capacities, divided by technology, in each selected country in 2018 is detailed in Table 1, Table 2, Table 3, Table 4, Table 5, Table 6 below (sources B-E). The methods used to calculate these estimates are described in more detail in Section 2.1. Data on the installation year of each power plant can be found in the country datasets published on Zenodo (see Appendix B).

**Table 2:** Estimated installed on-grid electricity generation capacity (MW) by technology type in selected countries in Africa in 2018 (sources B-D). ‘-’ denotes 0 estimated capacity.

	Estimated Installed Capacity (MW)												
Country	Biomass	Oil	Coal	Gas CCGT	Gas SCGT	Geothermal	Utility-scale Solar PV	Concentrating Solar Power	Large Hydropower (>100 MW)	Medium Hydropower (10–100 MW)	Small Hydropower (<10 MW)	Onshore Wind	Nuclear
Algeria	-	-	-	35738	143	-	54	25	251	24	-	-	-
Angola	-	351	-	-	420	-	14	-	849	71	-	-	-
Benin	-	20	-	139.1	-	-	-	-	-	-	-	-	-
Botswana	-	-	746	-	-	-	-	-	-	-	-	-	-
Burkina Faso	-	267	-	-	-	-	-	-	-	-	-	-	-
Burundi	-	-	-	-	-	-	-	-	-	49	-	-	-
Cameroon	-	168	-	450	200	-	-	-	550	171	-	-	-
Central African Republic	-	14	-	-	-	-	-	-	-	19	-	-	-
Chad	-	217	-	-	-	-	-	-	-	-	-	-	-
Republic of Congo	-	71.1	-	336	-	-	-	-	120	99	-	-	-
Côte D'Ivoire	-	-	-	716	504	-	-	-	549	50	-	-	-
Djibouti	-	107	-	-	-	-	-	-	-	-	-	-	-
Democratic Republic of Congo	-	13	-	-	25	-	-	-	2533	418	-	-	-
Egypt	-	1146	-	16546	294	-	25	20	2700	150	-	810	-
Equatorial Guinea	-	-	-	-	185	-	-	-	150	-	-	-	-
Eritrea	-	133	-	-	-	-	-	-	-	-	-	-	-
Eswatini	120	-	-	-	-	-	-	-	-	51	-	-	-
Ethiopia	-	-	-	-	-	8.5	23	-	3621	191	-	171	-
Gabon	-	16	-	306.9	-	-	-	-	160	170	-	-	-
Gambia	-	70	-	-	-	-	-	-	-	-	-	-	-
Ghana	-	-	-	1251	-	-	-	-	1598	-	-	-	-
Guinea	-	371	-	-	-	-	-	-	240	178	-	-	–
Guinea Bissau	-	18	-	-	-	-	-	-	-	-	-	-	-
Kenya	90	735	-	-	-	419	24	-	499	249	-	310	-
Lesotho	-	-	-	-	-	-	-	-	-	72	-	-	-
Liberia	-	121	-	-	-	-	-	-	-	60	-	-	-
Libya	-	191	-	6762	2120	-	-	-	-	-	-	-	-
Malawi	10	-	-	-	16	-	-	-	252	92	-	-	-
Mali	-	-	-	-	-	-	-	-	200	106	5	-	-
Mauritania	-	133	-	-	120	-	33	-	-	97	-	30	-
Morocco	-	338	2621	1666	-	-	-	690	1207	453	8	1266	-
Mozambique	-	-	-	-	-	-	-	-	2242	44	-	-	–
Namibia	-	60	120	-	-	-	27.4	-	240	92	-	-	-
Niger	-	124	38	-	-	-	-	-	-	-	-	-	-
Nigeria	-	-	-	5158	3261	-	17	-	2040	-	-	-	-
Rwanda	-	28	-	-	26	-	9	-	-	126	3	-	-
Senegal	25	856	-	-	-	-	11	-	120	-	-	-	-
Sierra Leone	-	76	-	-	-	-	-	-	-	50	-	-	-
Somalia	-	15.6	-	-	-	-	1	-	-	-	-	2	-
South Africa	224	2832	38295		376		1765	450	601	42	7	2032	1830
South Sudan	-	64.9	-	-	-	-	-	-	-	-	-	-	-
Sudan	191	926	-	319	-	-	-	-	2196	56	-	-	-
Tanzania	70	100	-	208	213	-	-	-	384	169	8	-	-
Togo	-	101	-	28	-	-	-	-	-	66	-	-	-
Tunisia	-	-	-	3559	145	-	22	-	-	57.8	8	245	-
Uganda	60	136	-	-	-	-	20	-	635	71.5	-	-	-
Zambia	39.8	170	300	-	-	-	-	-	2308	12	-	-	-
Zimbabwe	97	9.83	1117.5	-	-	-	-	-	680	-	-	-	-

**Table 3:** Estimated installed on-grid electricity generation capacity (MW) by technology type in S=selected countries in East Asia in 2018 (sources B-D) ‘-’ denotes 0 estimated capacity.

	Estimated Installed Capacity (MW)											
Country	Biomass	Oil	Coal	Gas CCGT	Gas SCGT	Geothermal	Utility-scale Solar PV	Concentrating Solar Power	Large Hydropower (>100 MW)	Medium Hydropower (10–100 MW)	Small Hydropower (<10 MW)	Onshore Wind
Cambodia	2	54	506	-	-	-	12	897	33	-	-	-
Indonesia	1740	751.7	34397	13799	1170	1404	-	4539.41	505	42	-	-
Laos	30	-	1876	-	-	-	-	4221.14	222	19	-	-
Malaysia	1040	178	14334	14324	626	-	262	4500	168	-	-	-
Myanmar	-	0	252.49	1448.6	35	-	12	2568.8	647	-	-	-
Papua New Guinea	-	361.9	-	-	186.2	56	-	-	271	4	-	-
Philippines	190	2709	10699	4237.5	100	1918.8	818	3393.9	208	1	220.1	-
South Korea	858	3255	38260	33583	118	-	3533	4827	374	62	768.9	23080
Taiwan	740	2632	18650	13561	-	-	842	3778	41	3	646.64	5216
Thailand	3230		5259	27132	-	-	1415	3639	148	-	210	-
Vietnam	255	970.7	14935	8201.5	-	-	-	13534	2957	266	287	-

**Table 4:** Estimated installed on-grid electricity generation capacity (MW) by technology type in selected countries in South America in 2018 (sources B-D) ‘-’ denotes 0 estimated capacity.

	Estimated Installed Capacity (MW)										
Country	Biomass	Oil	Coal	Gas CCGT	Gas SCGT	Geothermal	Utility-scale Solar PV	Concentrating Solar Power	Large Hydropower (>100 MW)	Medium Hydropower (10–100 MW)	Small Hydropower (<10 MW)
Argentina	660	1755.55	2634.54	13775.38	1362	3	9204	589	52	279.32	1764
Bolivia	150	-	-	1315.58	383	13	-	496.9	-	-	-
Brazil	12271.5	13403.2	4145.5	19287.36	718.6	6	88787	6917	1062	11378.2	1990
Chile	466.1	3365.18	4835	4878.89	72	618.15	5698	1460	165	909.18	-
Colombia	240	188	775.9	3151.06	-	-	11434	66	-	20	-
Ecuador	140	1693.3	-	146	1420.54	30	3089	167	-	20	-
Paraguay	40	-	-	-	-	-	8764	50	-	-	-
Peru	180	-	230.67	7373.13	75	96	4105.6	46	-	150	-
Uruguay	425.3	905.2	-	570.34	54	227	1538	-	-	1384	-
Venezuela	-	3570	-	10857	680	-	17560	105	-	30	-

**Table 5:** Estimated installed off-grid solar PV and hydropower capacity (MW) in selected countries in Africa in 2018 (source E) ‘-’ denotes 0 estimated capacity.

	Estimated Installed Capacity (MW)	
Country	Off-Grid Solar PV	Off-Grid Hydropower
Algeria	423	-
Angola	13.38	6.86
Benin	-	-
Botswana	1.61	-
Burkina Faso	27	-
Burundi	4.7	1.6
Cameroon	14.19	0.3
Central African Republic	0.3	0.2
Chad	0.17	-
Republic of Congo	0.57	-
Côte D'Ivoire	8.28	-
Djibouti	0.36	-
Democratic Republic of Congo	18.9	131.7
Egypt	50	-
Equatorial Guinea	-	-
Eritrea	10.16	-
Eswatini	0.8	1.7
Ethiopia	13.94	1.28
Gabon	1.4	0.29
Gambia	2	-
Ghana	7.59	-
Guinea	13.28	2.22
Guinea Bissau	1.17	-
Kenya	37.84	6.44
Lesotho	0.16	0.18
Liberia	2.58	4
Libya	5.11	0
Malawi	21.88	1.7
Mali	19.58	-
Mauritania	21.07	-
Morocco	22.9	-
Mozambique	15	1.06
Namibia	21.93	-
Niger	20.04	-
Nigeria	17.57	0.4
Rwanda	25.4	-
Senegal	12	-
Sierra Leone	4.25	4.89
Somalia	7.07	-
South Africa	-	7.19
South Sudan	0.55	-
Sudan	12.58	-
Tanzania	25.34	15.42
Togo	3	-
Tunisia	2.08	-
Uganda	28	5.4
Zambia	0.22	2.61
Zimbabwe	7.91	1.6

**Table 6:** Estimated installed off-grid solar PV and hydropower capacity (MW) in selected countries in East Asia in 2018 (source E). ‘-’ denotes 0 estimated capacity.

	Estimated Installed Capacity (MW)	
Country	Off-Grid Solar PV	Off-Grid Hydropower
Cambodia	1.94	-
Indonesia	45.08	14.85
Laos	1.63	2.01
Malaysia	7.44	0.45
Myanmar	47.54	6.89
Papua New Guinea	1.23	76.4
Philippines	1.16	20.59
South Korea	-	-
Taiwan	-	-
Thailand	-	-
Vietnam	5.26	43

**Table 7:** Estimated installed off-grid solar PV and hydropower capacity (MW) in selected countries in South America in 2018 (source E). ‘-’ denotes 0 estimated capacity.

	Estimated Installed Capacity (MW)	
Country	Off-Grid Solar PV	Off-Grid Hydropower
Argentina	0.62	19.58
Bolivia	5.54	9.2
Brazil	7.23	0.02
Chile	-	-
Colombia	1.53	5.32
Ecuador	2.02	26
Paraguay	0.06	-
Peru	53.58	177.65
Uruguay	2.27	-
Venezuela	3.4	0.81

### 1.2 Techno-economic data for electricity generation technologies

The techno-economic parameters of electricity generation technologies by region are presented in Table 8, Table 9 and 10, including costs, operational lives, efficiencies and average capacity factors. Two types of costs are considered here: capital costs, which are the initial investment costs for the electricity generation technology, and fixed costs, which are the fixed annual maintenance costs incurred when using the electricity generation technology, for example the costs of staffing the power plant or maintaining technical equipment. The efficiency of electricity generation technolologies is a measure of how much energy is lost in the conversion process to produce electricity, for example if a power plant is provided with two energy units of gas and produces one energy unit of electricity, with the rest of the energy lost as waste heat, the power plant would have an efficieny of 50%. Capacity factors are a measure of how often an electricity generation technology is producing over a given period of time, for example wind turbines are likely to have a lower capacity factor than gas power plants as wind turbines can only generate electricity when the wind conditions are suitable. Capacity factors for renewable technologies, including wind turbines, solar PV panels and hydropower plants, are dependent on their location as conditions vary with geography.

**Table 8:** Techno-economic parameters of electricity generation technologies in Africa (sources F, G, P).

Technology	Capital Cost ($/kW in 2020)	Fixed Cost ($/kW/yr in 2020)	Operational Life (years)	Efficiency (%)	Average Capacity Factor (%)
Biomass Power Plant	2500	75	30	35	50
Coal Power Plant	2500	78	35	37	85
Geothermal Power Plant	4000	120	25	80	79
Light Fuel Oil Power Plant	1200	35	25	35	80
Oil Fired Gas Turbine (SCGT)	1450	45	25	35	80
Gas Power Plant (CCGT)	1200	35	30	48	85
Gas Power Plant (SCGT)	700	20	25	30	85
Solar PV (Utility)	1378	18	24	100	Varies by country
Concentrating Solar Power without Storage	4058	41	30	100	23
Concentrating Solar Power with Storage	5797	58	30	100	26
Large Hydropower Plant (Dam) (>100MW)	3000	90	50	100	Varies by country
Medium Hydropower Plant (10-100MW)	2500	75	50	100	Varies by country
Small Hydropower Plant (<10MW)	3000	90	50	100	Varies by country
Onshore Wind	1489	60	25	100	Varies by country
Offshore Wind	3972	159	25	100	Varies by country
Nuclear Power Plant	6137	184	50	33	85
Light Fuel Oil Standalone Generator (1kW)	750	23	10	16	30
Solar PV (Distributed with Storage)	4320	86	24	100	Varies by country

**Table 9:** Techno-economic parameters of electricity generation technologies in East Asia (sources I-J).

Technology	Capital Cost ($/kW in 2020)	Fixed Cost ($/kW/yr in 2020)	Operational Life (years)	Efficiency	Average Capacity Factor
Biomass Power Plant	2750.0	69.0	25	0.38	0.7
Coal Power Plant	1300.0	52.0	60	0.3	0.75
Geothermal Power Plant	2500.0	100.0	50	0.1	0.7
Light Fuel Oil Power Plant	1200.0	18.0	50	0.4	0.25
Oil Fired Gas Turbine (SCGT)	1344.0	18.0	50	0.4	0.25
Gas Power Plant (CCGT)	1000.0	40.0	30	0.55	0.55
Gas Power Plant (SCGT)	784.0	23.0	30	0.35	0.55
Solar PV (Utility)	1160.0	15.08	30	1.0	Varies by country
Concentrating Solar Power with Storage	4965.31	120.0	35	0.33	0.3
Large Hydropower Plant (Dam) (>100 MW)	1539.0	46.17	40	1.0	Varies by country
Medium Hydropower Plant (10–100 MW)	1592.86	47.79	40	1.0	Varies by country
Small Hydropower Plant (<10 MW)	2162.0	64.86	40	1.0	Varies by country
Onshore Wind	2220.09	88.8	30	1.0	Varies by country
Offshore Wind	2876.21	115.05	30	1.0	Varies by country
Nuclear Power Plant	5500.0	138.0	60	0.33	0.83
Light Fuel Oil Standalone Generator (1 kW)	1500.0	38.0	20	0.42	0.4
Solar PV (Distributed with Storage)	2130.8	42.62	24	1.0	Varies by country

**Table 10:** Techno-economic parameters of electricity generation technologies in South America (sources J-K).

Technology	Capital Cost ($/kW in 2020)	Fixed Cost ($/kW/yr in 2020)	Operational Life (years)	Efficiency	Average Capacity Factor
Biomass Power Plant	1905.0	13.0	25	0.35	0.7
Coal Power Plant	2500.0	40.0	40	0.43	0.75
Geothermal Power Plant	3796.47	100.0	20	0.11	0.7
Light Fuel Oil Power Plant	1200.0	15.0	25	0.35	0.25
Oil Fired Gas Turbine (SCGT)	1400.0	25.0	25	0.35	0.25
Gas Power Plant (CCGT)	1260.0	20.0	30	0.57	0.55
Gas Power Plant (SCGT)	583.0	10.0	30	0.38	0.55
Solar PV (Utility)	1524.5	19.8	25	1.0	Varies by country
Concentrating Solar Power with Storage	5797.0	57.97	40	0.35	0.3
Large Hydropower Plant (Dam) (>100 MW)	2939.0	88.17	60	1.0	Varies by country
Medium Hydropower Plant (10–100 MW)	2500.0	75.0	60	1.0	Varies by country
Small Hydropower Plant (<10 MW)	3499.0	104.9	60	1.0	Varies by country
Onshore Wind	1375.6	55.0	30	1.0	Varies by country
Offshore Wind	3406.3	136.2	25	1.0	Varies by country
Nuclear Power Plant	6318.0	189.54	40	0.35	0.83
Light Fuel Oil Standalone Generator (1 kW)	750.0	23.0	20	0.42	0.4
Solar PV (Distributed with Storage)	4320.0	86.4	24	1.0	Varies by country

For countries in Africa, cost (capital and fixed), operational life and efficiency data were collected from reports by the International Renewable Energy Agency (IRENA) (sources F-H) and applied to all of Africa. These cost data include projected cost reductions for renewable energy technologies, expected to occur with increasing deployment and economies of scale,which are presented in Table 11. Cost (capital and fixed), operational life and efficiency data for countries in East Asia are based on reports by the International Renewable Energy Agency and the ASEAN Centre for Clean Energy (ACE) (sources I-J). Cost (capital and fixed), operational life and efficiency data for countries in South America are based on the data used in the South America Model Base (SAMBA) (source K). Where technologies were not included in SAMBA, namely diesel generation technologies, medium hydropower plants and decentralised solar PV with storage, costs were estimated based on costs in other regions. For countries in Asia and South America, projected cost reductions for renewable energy technologies were estimated by applying the cost reduction trends of IRENA for Africa (source F), published in 2021, to current Asia- and South America-specific cost estimates. The cost and performance of parameters of fossil electricity generation technologies are assumed constant over the modeling period. Only fixed power plant costs are considered in this analysis, which have been calculated to also capture variable operation and maintenance costs. Country-specific capacity factors for solar PV, onshore wind and hydropower technologies for every country were sourced from Renewables Ninja and the PLEXOS-World 2015 Model Dataset (sources B, L, M). Country-specific capacity factors for offshore wind were sourced from the TEMBA dataset (source N) for countries in Africa and an National Renewable Energy Laboratory (NREL) dataset for countries in East Asia and South America (source O). Regional capacity factor estimates for other technologies were sourced from the IRENA (sources G, J) for Africa, SAMBA for South America (source K), and IRENA and ACE for Asia (source I). Average capacity factors were calculated for each technology and presented below, with daytime (6 am–6 pm) averages presented for solar PV technologies. For more information on the capacity factor data, refer to Section 2.1.

**Table 11:** Projected costs of renewable energy technologies for in Africa selected years to 2050 (sources F, P).

	Capital Cost ($/kW)					
Renewable Energy Technology	2015	2020	2025	2030	2040	2050
Biomass Power Plant	2500	2500	2500	2500	2500	2500
Geothermal Power Plant	4000	4000	4000	4000	4000	4000
Solar PV (Utility)	2165	1378	984	886	723	723
Concentrating Solar Power without Storage	6051	4058	3269	2634	2562	2562
Concentrating Solar Power with Storage	8645	5797	4670	3763	3660	3660
Large Hydropower Plant (Dam) (>100 MW)	3000	3000	3000	3000	3000	3000
Medium Hydropower Plant (10–100 MW)	2500	2500	2500	2500	2500	2500
Small Hydropower Plant (<10 MW)	3000	3000	3000	3000	3000	3000
Onshore Wind	1985	1489	1191	1087	933	933
Offshore Wind	5000	3972	3021	2450	2275	2100
Solar PV (Distributed with Storage)	6840	4320	3415	2700	2091	2091

**Table 12:** Projected costs of renewable energy technologies in East Asia for selected years to 2050 (sources F, I, J, P).

	Capital Cost ($/kW)					
Renewable Energy Technology	2015	2020	2025	2030	2040	2050
Biomass Power Plant	2750.0	2750.0	2750.0	2750.0	2750.0	2750.0
Solar PV (Utility)	1822.5	1160.0	828.33	745.83	608.62	608.62
Concentrating Solar Power with Storage	7404.71	4965.31	4000.0	3223.13	3134.9	3134.9
Large Hydropower Plant (Dam) (>100 MW)	1539.0	1539.0	1539.0	1539.0	1539.0	1539.0
Medium Hydropower Plant (10–100 MW)	1592.86	1592.86	1592.86	1592.86	1592.86	1592.86
Small Hydropower Plant (<10 MW)	2162.0	2162.0	2162.0	2162.0	2162.0	2162.0
Onshore Wind	2959.63	2220.09	1775.78	1620.71	1391.1	1391.1
Offshore Wind	3620.25	2876.21	2187.28	1773.92	1647.21	1520.5
Solar PV (Distributed with Storage)	3502.0	2130.8	1880.8	1755.8	1690.8	1625.8

**Table 13:** Projected costs of renewable energy technologies in South America for selected years to 2050 (sources F, K, P).

	Capital Cost ($/kW)					
Renewable Energy Technology	2015	2020	2025	2030	2040	2050
Biomass Power Plant	1905.0	1905.0	1905.0	1905.0	1905.0	1905.0
Solar PV (Utility)	1898.79	1791.02	1683.26	1575.49	1359.96	1144.43
Concentrating Solar Power with Storage	8652.93	5797.0	4670.0	3763.0	3660.0	3660.0
Large Hydropower Plant (Dam) (>100 MW)	2939.0	2939.0	2939.0	2939.0	2939.0	2939.0
Medium Hydropower Plant (10–100 MW)	2500.0	2500.0	2500.0	2500.0	2500.0	2500.0
Small Hydropower Plant (<10 MW)	3499.0	3499.0	3499.0	3499.0	3499.0	3499.0
Onshore Wind	1620.0	1582.33	1544.65	1506.98	1431.63	1356.28
Offshore Wind	4104.0	3928.19	3752.37	3576.56	3224.93	2873.3
Solar PV (Distributed with Storage)	6840.0	4320.0	3415.0	2700.0	2091.0	2091.0

**Table 14:** Estimated average capacity factors in selected countries in Africa (sources B, C, L, M, Q).

Country	Hydropower	Solar PV	Onshore Wind	Offshore Wind
Algeria	0.11	0.35	0.21	0.37
Angola	0.52	0.32	0.11	0.12
Benin	0.36	0.27	0.13	0.13
Botswana	0.23	0.35	0.21	n/a
Burkina Faso	0.35	0.37	0.17	n/a
Burundi	0.42	0.28	0.06	n/a
Cameroon	0.6	0.31	0.07	0.1
Central African Republic	0.7	0.28	0.08	n/a
Chad	0.43	0.33	0.26	n/a
Republic of Congo	0.47	0.25	0.03	0.09
Côte D'Ivoire	0.33	0.17	0.09	0.1
Djibouti	0.41	0.29	0.21	0.36
Democratic Republic of Congo	0.34	0.26	0.06	n/a
Egypt	0.54	0.36	0.22	0.4
Equatorial Guinea	0.19	0.23	0.03	0.08
Eritrea	0.41	0.3	0.16	0.46
Eswatini	0.42	0.32	0.14	n/a
Ethiopia	0.41	0.37	0.18	0.48
Gabon	0.55	0.25	0.04	0.1
Gambia	0.41	0.28	0.14	0.14
Ghana	0.58	0.27	0.1	0.1
Guinea	0.43	0.35	0.08	0.11
Guinea Bissau	0.41	0.26	0.11	0.15
Kenya	0.48	0.32	0.21	0.45
Lesotho	0.69	0.36	0.15	n/a
Liberia	0.6	0.27	0.07	0.37
Libya	0.41	0.35	0.29	0.4
Malawi	0.54	0.44	0.16	n/a
Mali	0.54	0.37	0.22	n/a
Mauritania	0.41	0.37	0.35	0.41
Morocco	0.13	0.37	0.36	0.33
Mozambique	0.68	0.3	0.18	0.3
Namibia	0.59	0.41	0.15	n/a
Niger	0.36	0.37	0.27	n/a
Nigeria	0.36	0.34	0.15	0.37
Rwanda	0.38	0.33	0.06	n/a
Senegal	0.41	0.41	0.17	0.37
Sierra Leone	0.36	0.26	0.08	0.37
Somalia	0.41	0.29	0.52	0.58
South Africa	0.23	0.38	0.25	0.36
South Sudan	0.5	0.19	0.15	n/a
Sudan	0.43	0.26	0.22	0.28
Tanzania	0.47	0.38	0.14	0.3
Togo	0.23	0.26	0.12	0.37
Tunisia	0.13	0.33	0.21	0.41
Uganda	0.54	0.31	0.08	n/a
Zambia	0.63	0.32	0.21	n/a
Zimbabwe	0.68	0.34	0.2	n/a

**Table 15:** Estimated average capacity factors in selected countries in East Asia (sources B, C, L, M, O).

Country	Hydropower	Solar PV	Onshore Wind	Offshore Wind
Cambodia	0.31	0.33	0.09	0.19
Indonesia	0.32	0.4	0.03	0.2
Laos	0.55	0.28	0.08	n/a
Malaysia	0.35	0.27	0.04	0.18
Myanmar	0.45	0.34	0.09	0.21
Papua New Guinea	0.38	0.27	0.11	0.24
Philippines	0.26	0.19	0.2	0.23
South Korea	0.2	0.25	0.19	0.24
Taiwan	0.19	0.24	0.27	0.34
Thailand	0.25	0.38	0.16	0.19
Vietnam	0.49	0.23	0.15	0.27

**Table 16:** Estimated average capacity factors in selected countries in South America (sources B, C, L, M, O).

Country	Hydropower	Solar PV	Onshore Wind	Offshore Wind
Argentina	0.42	0.38	0.32	0.45
Bolivia	0.54	0.2	0.13	n/a
Brazil	0.62	0.29	0.33	0.24
Chile	0.49	0.5	0.28	0.4
Colombia	0.5	0.24	0.41	0.27
Ecuador	0.52	0.3	0.07	0.18
Paraguay	0.72	0.29	0.2	n/a
Peru	0.66	0.44	0.29	n/a
Uruguay	0.52	0.17	0.27	0.31
Venezuela	0.63	0.28	0.2	0.3

### 1.3 Techno-economic data for electricity transmission and distribution

Transmission and distribution systems are used to transport electricity produced by on-grid electricity generation technologies, such as gas power plants, to sites of demand, such as homes and businesses. Transmission systems are used for transport over longer distances at higher voltages, while distribution systems transport electricity over shorter distances at lower voltages. The techno-economic parameters of transmission and distribution technologies are taken from The Reference Case scenario of The Electricity Model Base for Africa (TEMBA) (source Q) for countries in Africa. This gives estimated transmission and distribution efficiencies projected to 2050, and estimated costs and operational lives. The efficiency of transmission and distribution systems is a measure of how much energy is lost when transporting the electricity, for example as waste heat. For countries in Asia, combined losses in electricity transmission and distribution are estimated based on an International Energy Agency (IEA) dataset presented by Index Mundi (source R), which gives estimated combined losses in 2014. It was then assumed that combined losses would be reduced to 5% by 2050, falling linearly, due to assumed improvements in the technical operation of these systems and reduced non-technical losses, such as those due to power theft. The combined costs of power transmission and distribution are estimated based on a report by the Economic Research Institute for ASEAN and East Asia (ERIA) (source S), which gives cost estimates for several real-life projects in ASEAN. For countries in South America, the efficiencies and costs of power transmission and distribution were taken from the SAMBA dataset (source K), which gives estimated efficiencies by country, including projections to 2063. The estimated combined efficiencies of transmission and distribution in each included country are presented in the following tables.

**Table 17:** Estimated combined efficiency of transmission and distribution in selected countries in Africa in 2020, 2030 & 2050 (source Q).

	Estimated combined efficiency of transmission & distribution		
Country	2020 (%)	2030 (%)	2050 (%)
Algeria	71.3	74.1	77.9
Angola	89.3	90.3	90.3
Benin	77.9	78.9	80.8
Botswana	81.6	82.6	85.4
Burkina Faso	49.4	51.3	55.1
Burundi	86.5	87.4	89.3
Cameroon	77.0	77.9	79.8
Central African Republic	77.0	77.9	79.8
Chad	77.0	77.9	79.8
Republic of Congo	52.3	54.2	58.0
Côte D'Ivoire	80.8	81.7	81.7
Djibouti	77.9	78.9	80.8
Democratic Republic of Congo	87.5	87.4	89.3
Egypt	87.4	88.3	90.2
Equatorial Guinea	74.1	76.0	79.8
Eritrea	80.8	81.7	83.6
Eswatini	90.3	90.3	90.3
Ethiopia	87.4	88.3	90.2
Gabon	58.9	60.8	64.6
Gambia	77.9	79.8	81.7
Ghana	77.0	77.9	79.8
Guinea	90.3	90.3	92.2
Guinea Bissau	43.7	45.6	50.4
Kenya	81.7	83.6	88.4
Lesotho	83.6	88.4	89.3
Liberia	71.3	74.1	77.9
Libya	67.5	69.4	73.2
Malawi	78.9	79.8	81.7
Mali	79.8	79.8	81.7
Mauritania	59.9	61.8	65.6
Morocco	86.5	87.4	89.3
Mozambique	82.7	84.6	89.3
Namibia	90.2	91.2	93.1
Niger	74.1	76.0	79.8
Nigeria	86.5	87.4	89.3
Rwanda	66.5	75.1	78.9
Senegal	86.5	87.4	89.3
Sierra Leone	54.2	56.1	63.7
Somalia	53.2	55.1	62.7
South Africa	91.2	92.2	92.2
South Sudan	90.3	90.3	92.2
Sudan	90.3	90.3	91.2
Tanzania	83.6	84.6	87.4
Togo	86.5	87.4	89.3
Tunisia	83.6	84.6	87.4
Uganda	82.7	83.6	86.5
Zambia	86.4	87.4	89.3

**Table 18:** Estimated combined efficiency of transmission and distribution in selected countries in Asia in 2020, 2030 & 2050 (source R).

	Estimated combined efficiency of transmission & distribution		
Country	2020 (%)	2030 (%)	2050 (%)
Cambodia	80.0	85.0	95.0
Indonesia	91.0	93.0	95.0
Laos	91.0	92.0	95.0
Malaysia	94.0	95.0	95.0
Myanmar	82.0	86.0	95.0
Papua New Guinea	90.0	92.0	95.0
Philippines	91.0	93.0	95.0
South Korea	86.0	89.0	95.0
Taiwan	92.0	93.0	95.0
Thailand	94.0	94.3	95.0
Vietnam	91.0	93.0	95.0

**Table 19:** Estimated combined efficiency of transmission and distribution in selected countries in South America in 2020, 2030 & 2050 (source K).

	The estimated combined efficiency of transmission & distribution		
Country	2020 (%)	2030 (%)	2050 (%)
Argentina	87.4	89.3	90.2
Bolivia	86.5	89.3	91.2
Brazil	83.7	86.5	89.3
Chile	92.2	92.2	93.1
Colombia	88.4	90.2	92.2
Ecuador	89.3	91.2	92.2
Paraguay	75.1	79.7	87.4
Peru	90.3	92.2	92.2
Uruguay	84.6	87.4	90.2
Venezuela	71.6	77.1	85.5

### 1.4 Techno-economic data for refineries

Refineries are used to convert crude oil into useful fuels such as gasoline and diesel. Some countries have domestic refinery capacity, meaning they can process domestically-produced or imported crude oil, while others rely on importing oil-based fuels. Domestic refinery capacity in each country is sourced from the McKinsey Refinery Reference Desk (source T). In the example OSeMOSYS model, two oil refinery technologies were made available for investment in the future, each producing different ratios of Heavy Fuel Oil (HFO) and Light Fuel Oil (LFO). Heavy fuel oils are more viscous than lighter fuel oils such as gasoline. The techno-economic data for the two refinery technologies considered are shown in Table 23.

**Table 20:** Estimated domestic refinery capacity for selected countries in Africa (source T).

Country	Estimated Refinery Capacity (tb/d)
Algeria	671
Angola	65
Cameroon	70
Chad	20
Republic of Congo	21
Côte D'Ivoire	84
Egypt	800
Gabon	24
Ghana	45
Liberia	15
Libya	380
Morocco	200
Niger	20
Nigeria	445
Senegal	25
Sierra Leone	5
South Africa	545
Sudan	147
Tunisia	34
Zambia	12

**Table 21:** Estimated domestic refinery capacity for selected countries in East Asia (source T).

Country	Estimated Refinery Capacity (tb/d)
Indonesia	1147
Malaysia	915
Myanmar	57
Papua New Guinea	37
Philippines	292
South Korea	3504
Taiwan	1230
Thailand	1288
Vietnam	336

**Table 22:** Estimated domestic refinery capacity for selected countries in South America (source T).

Country	Estimated Refinery Capacity (tb/d)
Argentina	651
Bolivia	61
Brazil	2242
Chile	236
Colombia	403
Ecuador	176
Peru	211
Paraguay	8
Uruguay	50
Venezuela	1303

**Table 23:** Techno-economic parameters for refinery technologies (sources Q, U).

Technology	Capital Cost ($/kW in 2020)	Variable Cost ($/GJ in 2020)	Operational Life (years)	Output Ratio
Crude Oil Refinery Option 1	24.1	0.71775	35	0.9 LFO: 0.1 HFO
Crude Oil Refinery Option 2	24.1	0.71775	35	0.8 LFO: 0.2 HFO

### 1.5 Fuel prices

Assumed costs are provided for both imported and domestically-extracted fuels, with fuel price projections up to 2050 presented below. These are generic estimates based on an international oil price forecast (source V) and cost estimates for Africa (source G), Asia Pacific (sources W-Y), and South America (sources K, V, Z). A detailed explanation of how these estimates were sourced is provided in Section 2.2.

**Table 24:** Fuel price projections to 2050 for countries in Africa (sources G, V).

	Fuel Price ($/GJ)					
Commodity	2015	2020	2025	2030	2040	2050
Crude Oil Imports	13.1	12.2	12.8	14.3	16.9	19.5
Crude Oil Extraction	12.0	11.1	11.6	13.0	15.4	17.8
Biomass Imports	1.8	1.8	1.8	1.8	1.8	1.8
Biomass Extraction	1.6	1.6	1.6	1.6	1.6	1.6
Coal Imports	4.9	5.1	5.3	5.5	5.9	5.9
Coal Extraction	3.3	3.4	3.5	3.6	3.8	3.8
Light Fuel Oil Imports	15.9	14.7	15.4	17.3	20.4	23.6
Heavy Fuel Oil Imports	9.6	8.9	9.3	10.4	12.3	14.2
Natural Gas Imports	8.6	8.6	9.5	10.3	11.0	11.0
Natural Gas Extraction	7.1	7.1	7.8	8.5	9.9	9.9

**Table 25:** Fuel price projections to 2050 for countries in Asia (sources W-Y).

	Fuel Price ($/GJ)					
Commodity	2015	2020	2025	2030	2040	2050
Crude Oil Imports	6.27	13.95	15.12	16.29	19.84	21.33
Crude Oil Extraction	5.7	12.68	13.75	14.81	18.03	19.39
Biomass Imports	5.55	5.55	5.55	5.55	5.55	5.55
Biomass Extraction	1.34	1.34	1.34	1.34	1.34	1.34
Coal Imports	2.38	3.03	3.09	3.15	3.53	3.61
Coal Extraction	2.16	2.72	2.77	2.82	3.18	3.25
Light Fuel Oil Imports	6.83	15.21	16.49	17.77	21.64	23.26
Heavy Fuel Oil Imports	5.99	13.3	14.43	15.55	18.94	20.35
Natural Gas Imports	5.71	9.98	10.17	10.37	10.72	10.75
Natural Gas Extraction	5.16	8.98	9.16	9.34	9.65	9.67

**Table 26:** Fuel price projections to 2050 for countries in South America (sources K, V, Z).

	Fuel Price ($/GJ)					
Commodity	2015	2020	2025	2030	2040	2050
Crude Oil Imports	13.14	12.2	12.76	14.27	16.9	19.52
Crude Oil Extraction	11.95	11.09	11.6	12.97	15.36	17.75
Biomass Imports	6.16	6.16	6.16	6.16	6.16	6.16
Biomass Extraction	5.6	5.6	5.6	5.6	5.6	5.6
Coal Imports	3.2	3.55	3.64	3.73	3.9	4.26
Coal Extraction	2.91	3.23	3.31	3.39	3.55	3.87
Light Fuel Oil Imports	15.89	14.75	15.43	17.25	20.43	23.61
Heavy Fuel Oil Imports	9.56	8.87	9.28	10.38	12.29	14.2
Natural Gas Imports	3.76	4.65	5.54	6.43	8.22	10.01
Natural Gas Extraction	3.41	4.22	5.04	5.85	7.48	9.1

### 1.6 Emission factors

Electricity generation technologies fuelled by fossil fuels emit several greenhouse gases throughout their operational lifetime, including carbon dioxide, methane, and nitrous oxides. In these analyses and data kits, only carbon dioxide emissions are considered. These are accounted for using carbon dioxide emission factors assigned to each fuel rather than each power generation technology. The assumed emission factors are presented in Table 27.

**Table 27:** Fuel-specific CO2 emission factors (source AA).

Fuel	CO_2_ Emission Factor (kg CO_2_/GJ)
Crude oil	73.3
Biomass	100
Coal	94.6
Light Fuel Oil	69.3
Heavy Fuel Oil	77.4
Natural Gas	56.1

### 1.7 Renewable and fossil fuel reserves

Table 28, Table 29, Table 30, Table 31, Table 32, Table 33 show estimated domestic renewable energy potentials and fossil fuel reserves respectively by country. Sources used for each region are described in Section 2.3 and can be found in the external country-specific datasets produced for each country (see Appendix B).

**Table 28:** Estimated renewable energy potentials for Selected Countries in Africa (sources G, AB-AD; see individual country datasets in Appendix B for further detail). ‘-’ denotes 0 estimated potential.CF refers to capacity factor.

Country	Solar PV (TWh/yr)	Concentrating Solar Power (TWh/yr)	Wind (CF 20%, TWh/yr)	Wind (CF 30%, TWh/yr)	Wind (CF 40%, TWh/yr)	Hydropower (>10MW, MW)	Small Hydropower (<10MW, MW)	Geothermal (MW)
Algeria	27904	26530	30155	2535.9	153.4	-	-	-
Angola	13319	9786	202	-	-	7209	600	-
Benin	3898	-	405	-	-	436	69.9	-
Botswana	13764	13070	9793	303	-	-	1	-
Burkina Faso	7742	-	4154	7.5	-	133	17	-
Burundi	888	786	-	-	-	1700	61	-
Cameroon	10105	3706	979	15.9	-	23000	970	-
Central African Republic	5284	3471	79	-	-	2000	41	-
Chad	10506	10284	9165	1519.4	578.3	-	-	-
Republic of Congo	6778	2	-	-	-	2500	65	-
Côte D'Ivoire	10325	221	430	-	-	1764	45.7	-
Djibouti	947	852	934	149.1	77.3	-	-	-
Democratic Republic of Congo	22862	12439	2173	41.4	-	22573	101	-
Egypt	32218	26605	36601	6185	572.9	3664	51.7	-
Equatorial Guinea	706	-	-	-	-	1300	7.5	-
Eritrea	4775	4349	3154	412.4	129.1	-	-	-
Eswatini	572	559	476	9.7	-	62	16.2	-
Ethiopia	27154	22959	14838	3002	1981	45000	1500	5000
Gabon	5402	6	-	-	-	6000	7.8	-
Gambia	474	316	173	1.3	-	-	12	-
Ghana	7644	229	606	2.4	-	1887	17.4	-
Guinea	5204	467	2	-	-	5515	198	-
Guinea Bissau	1493	906	124	-	-	184	-	-
Kenya	23046	15399	22746	4446.4	1749.6	6000	3000	10000
Lesotho	938	1122	599	40.1	3.7	263	38.2	-
Liberia	667	-	-	-	-	971	56.4	-
Libya	13979	11823	21649	5149.5	1079.5	-	-	-
Malawi	5210	4474	1986	262.1	42.4	892	150	-
Mali	7906	-	1923	-	-	489	28.4	-
Mauritania	7990	4988	11822	2940.5	1337.8	-	-	-
Morocco	15155	15127	11297	1458.8	851	-	54	-
Mozambique	22024	16851	10805	395.9	5.2	5269	1000	-
Namibia	26183	29716	15196	497	4.9	600	120	-
Niger	15669	8829	14628	1262	55.8	359	8	-
Nigeria	32456	-	10045	95.3	-	5650	735	-
Rwanda	892	789	-	-	-	500	24.8	700
Senegal	7519	1537	5454	323.6	3	1400	-	-
Sierra Leone	1499	197	-	-	-	805	12.9	-
Somalia	25687	13156	43539	10616.4	8893.3	-	4.6	-
South Africa	42243	43275	41195	6076.3	1559.1	655	247	-
South Sudan	29272	25807	20553	3279	982	2927	24.7	-
Sudan	58544	51614	41101	6558	2947.1	4860	63.2	400
Tanzania	38804	31482	18456	2295.2	789.2	3800	480	650
Togo	1257	-	79	-	-	108	144	-
Tunisia	4645	2045	6842	1244	226.5	-	56	-
Uganda	9470	8582	815	100.7	23.8	4500	200	450
Zambia	17894	15691	13229	1145	15.6	6051	62	-
Zimbabwe	15864	11874	12137	1000.3	47.3	1850	120	-

**Table 29:** Estimated renewable energy potentials for selected countries in Asia (source O, AD-AG; see individual country datasets in Appendix B for further detail) ‘-’ denotes 0 estimated potential.

Country	Solar Resource (TWh/yr)	Concentrating Solar Power (TWh/yr)	Onshore Wind (TWh/yr)	Offshore Wind (TWh/yr)	Hydropower (>10MW, MW)	Small Hydropower (<10MW, MW)	Geothermal (MW)
Cambodia	545	-	550.35	350.3	10000	300	-
Indonesia	1613.1 (at LCOE <$150/MWh)	-	100 (at LCOE <$150/MWh)	12992.3	41436	12800	26150
Laos	179.79	-	14.02	-	26600	50.4	-
Malaysia	2646.7 (at LCOE <$150/MWh)	0.41	3 (at LCOE <$150/MWh)	1309.6	24970.5	39.5	273.25
Myanmar	1940	-	1424.5	2040.87	40400	231	4400
Papua New Guinea	1243	-	697.38	1792.05	4000	153	800
Philippines	2795 (at LCOE <$150/MWh)	-	442.8 (at LCOE <$150/MWh)	1097.03	10500	2021	4000
South Korea	251	-	519.16	301.71	15062	1500	-
Taiwan	36.1 (at LCOE <$150/MWh)	-	1888.6 (at LCOE <$150/MWh)	73	25700	714	-
Thailand	15575.5 (at LCOE <$150/MWh)	-	412.9 (at LCOE <$150/MWh)	2001.83	4542	700	6.6
Vietnam	3718.9 (at LCOE <$150/MWh)	-	636.1 (at LCOE <$150/MWh)	4553.93	16500	2887	400

**Table 30:** Estimated renewable energy potentials for selected countries in South America (sources K, O, AD, AG; see individual country datasets in Appendix B for further detail). ‘-’ denotes 0 estimated potential.

Country	Solar Resource (Twh/yr)	Onshore Wind (Twh/yr)	Offshore Wind (TWh/yr)	Hydropower (MW)	Small Hydropower (MW)	Geothermal (MW)
Argentina	7854	17900	10337.7	39970	430	2010
Bolivia	3220	1385.5	-	39800	200	2490
Brazil	24992	22215.5	7554.97	260093	-	-
Chile	1973	2081.5	2205.89	23043	2113	2350
Colombia	2888	1496.16	537.6	68000	25000	2210
Ecuador	607	136.98	73.19	24853.4	296.6	1700
Paraguay	1112	1258.39	-	12429.7	86.3	-
Peru	3577	594.37	484.79	58937	-	2990
Uruguay	480	1278.61	934.53	1607.2	207.8	-
Venezuela	2587	2958.28	1624.46	45952	48	910

**Table 31:** Estimated fossil fuel reserves for selected countries in Africa (sources AH-AI). ‘-’ denotes 0 estimated reserves.

Country	Total Recoverable Coal (mil. short tons, 2017)	Crude Oil Proven Reserves (billion barrels, 2019)	Natural Gas Proven Reserves (trillion cubic feet, 2019)
Algeria	65.04	12.2	159.05
Angola	-	8.38	14.91
Benin	-	0.01	-
Botswana	172.72	-	-
Burkina Faso	-	-	-
Burundi	-	-	-
Cameroon	-	0.2	4.77
Central African Republic	3.31	-	-
Chad	-	1.5	-
Republic of Congo	-	1.6	3.2
Cote D'Ivoire	-	0.1	1
Djibouti	-	-	-
Democratic Republic of Congo	97	0.18	0.04
Egypt	17.67	3.3	63
Equatorial Guinea	-	1.1	5.12
Eritrea	-	-	-
Eswatini	158.83	-	-
Ethiopia	-	-	0.88
Gabon	-	2	-
Gambia	-	-	-
Ghana	-	0.66	0.8
Guinea	-	-	-
Guinea Bissau	-	-	-
Kenya	-	-	-
Lesotho	-	-	-
Liberia	-	-	-
Libya	-	48.36	53.14
Malawi	2.2	-	-
Mali	-	-	-
Mauritania	-	0.02	15
Morocco	15.43	-	0.05
Mozambique	1975.34	-	100
Namibia	-	-	2.2
Niger	6.61	0.15	-
Nigeria	379.19	36.18	198.71
Rwanda	-	-	2
Senegal	-	-	-
Sierra Leone	-	-	-
Somalia	-	-	-
South Africa	34722.77	0.02	-
South Sudan	-	-	-
Sudan	-	5	3
Tanzania	296.52	-	0.23
Togo	-	-	-
Tunisia	-	0.43	2.3
Uganda	-	2.5	0.5
Zambia	49.66	-	-
Zimbabwe	553.36	-	-

**Table 32:** Estimated fossil fuel reserves for selected countries in Asia (sources W, AJ). ‘-’ denotes 0 estimated reserves.

Country	Coal (Million tonnes)	Crude Oil (Billion barrels)	Natural Gas (Trillion cubic metres)
Cambodia	-	0.03	-
Indonesia	22598	3.2	2.9
Laos	261.7	-	-
Malaysia	-	3.6	2.7
Myanmar	6.61	0.05	0.28
Papua New Guinea	-	0.2	0.19
Philippines	491	0.04	3.4
South Korea	315	-	0.01
Taiwan	-	-	-
Thailand	1063	0.35	0.2
Vietnam	3360	4.4	0.65

**Table 33:** Estimated fossil fuel reserves for selected countries in South America (sources K, AK). ‘-’ denotes 0 estimated reserves.

Country	Coal (Million short tons)	Crude Oil (billion barrels)	Natural Gas (Trillion cubic feet)
Argentina	600	2.8	12.2
Bolivia	-	0.2	10.3
Brazil	7300	15	13.4
Chile	200	0.2	-
Colombia	7400	2.4	3.7
Ecuador	-	8.2	-
Paraguay	-	-	-
Peru	-	0.6	12.4
Uruguay	-	-	-
Venezuela	500	298	223.8

### 1.8 Electricity demand projection

**Fig. 1:**
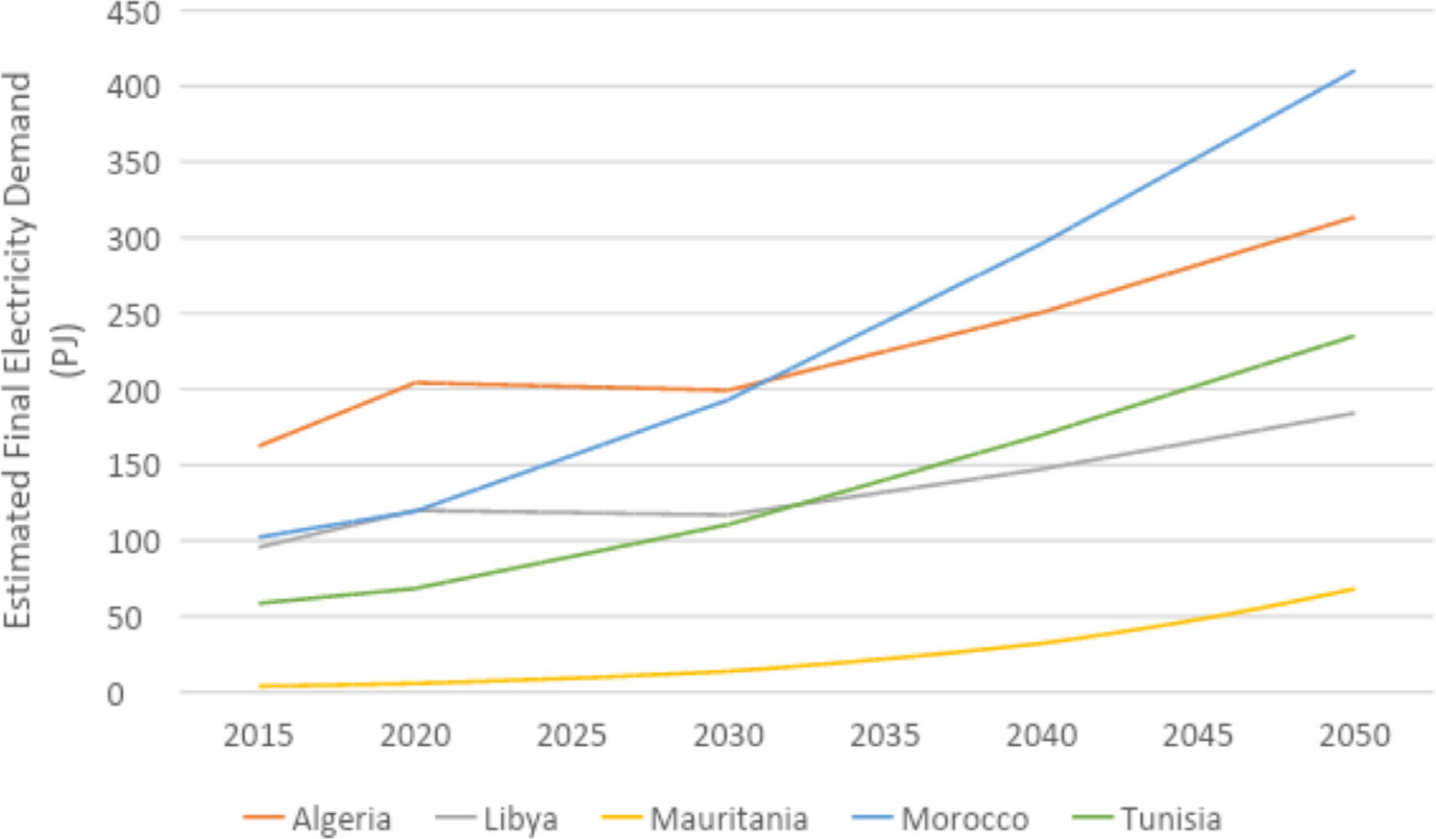
Final electricity demand projection (PJ) for countries in the North Africa Power Pool (source N).

**Fig. 2:**
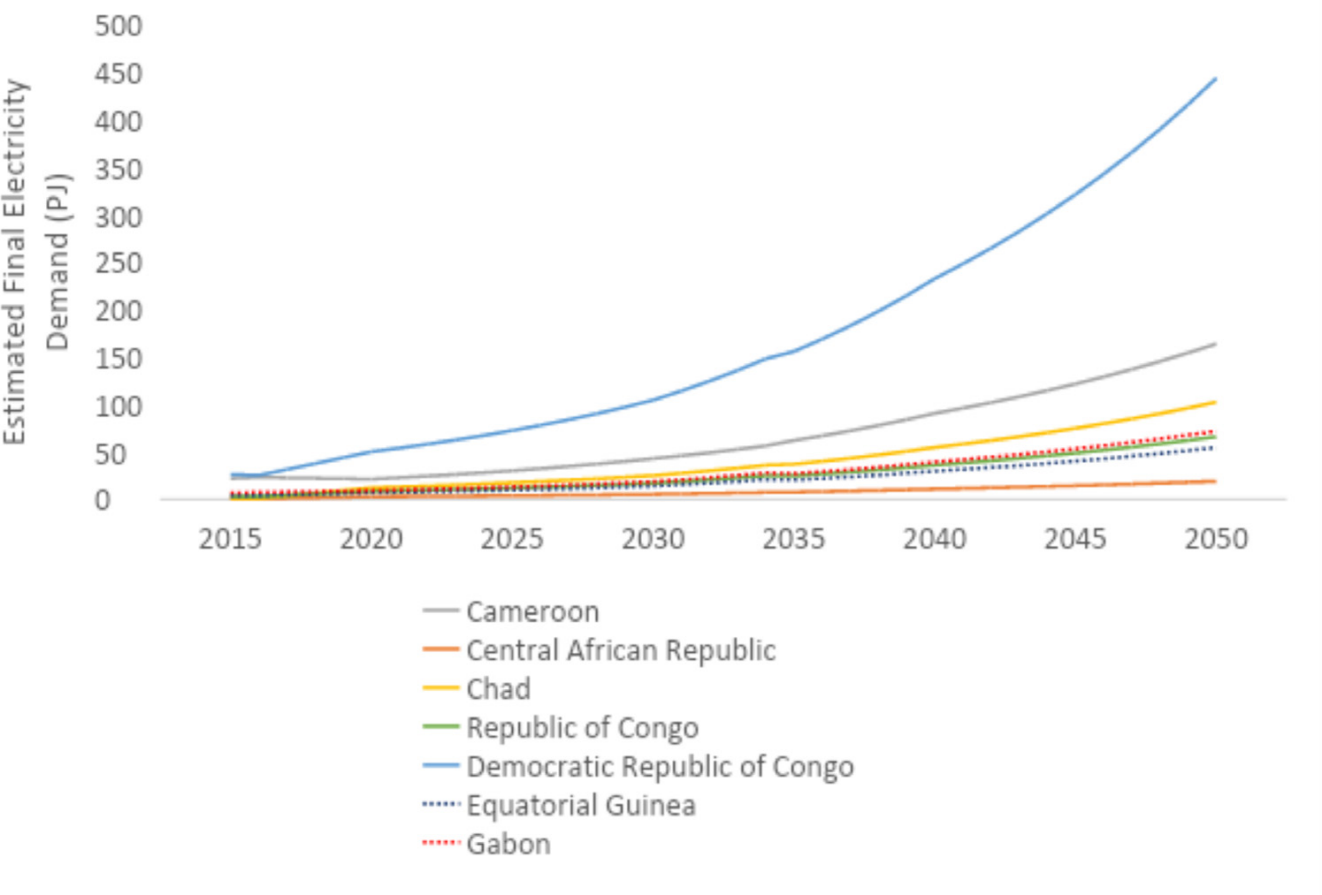
Final electricity demand projection (PJ) for countries in the Central Africa Power Pool (source N).

**Fig. 3:**
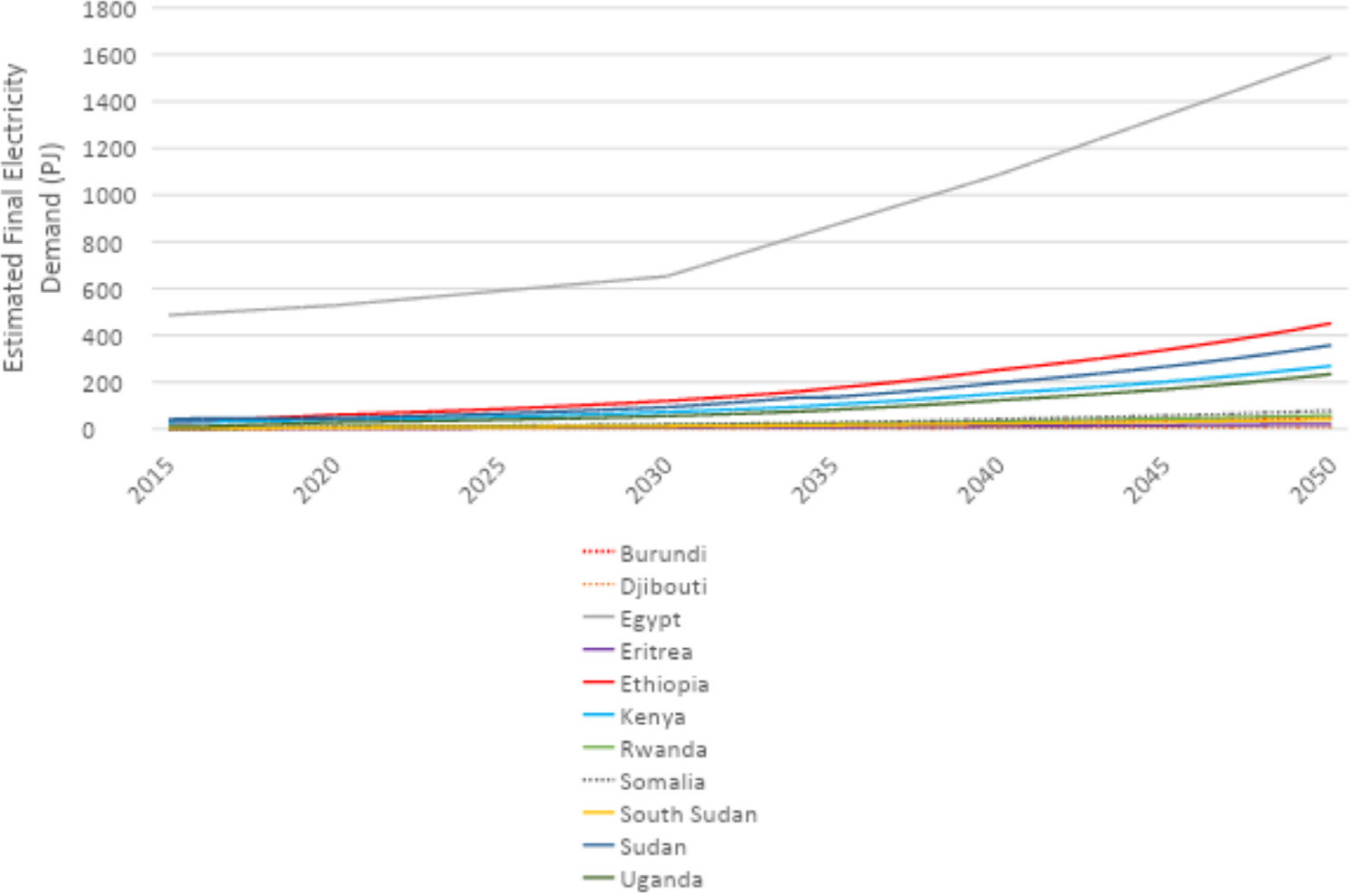
Final electricity demand projection (PJ) for countries in the East Africa Power Pool (source N).

**Fig. 4:**
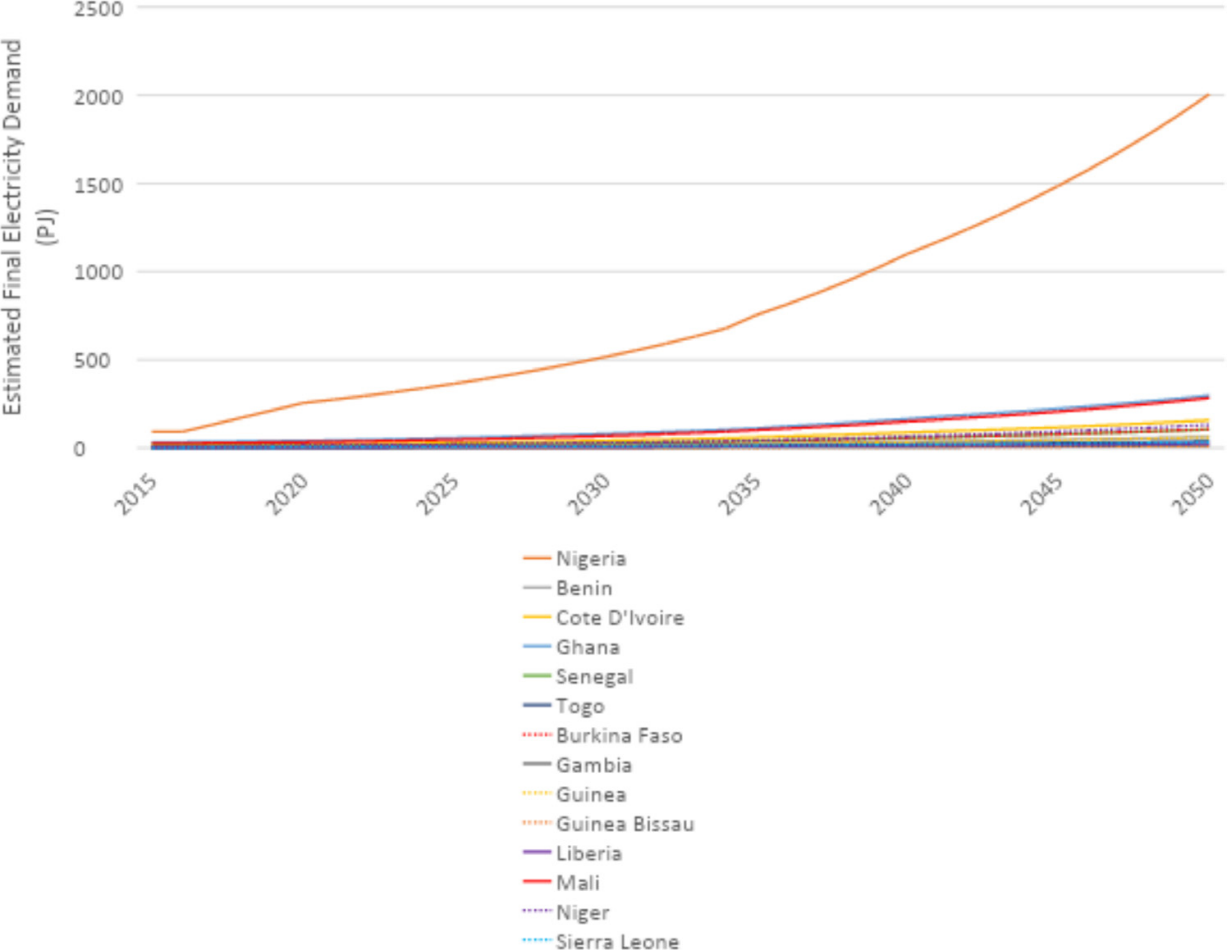
Final electricity demand projection (PJ) for countries in the West Africa Power Pool (source N).

**Fig. 5:**
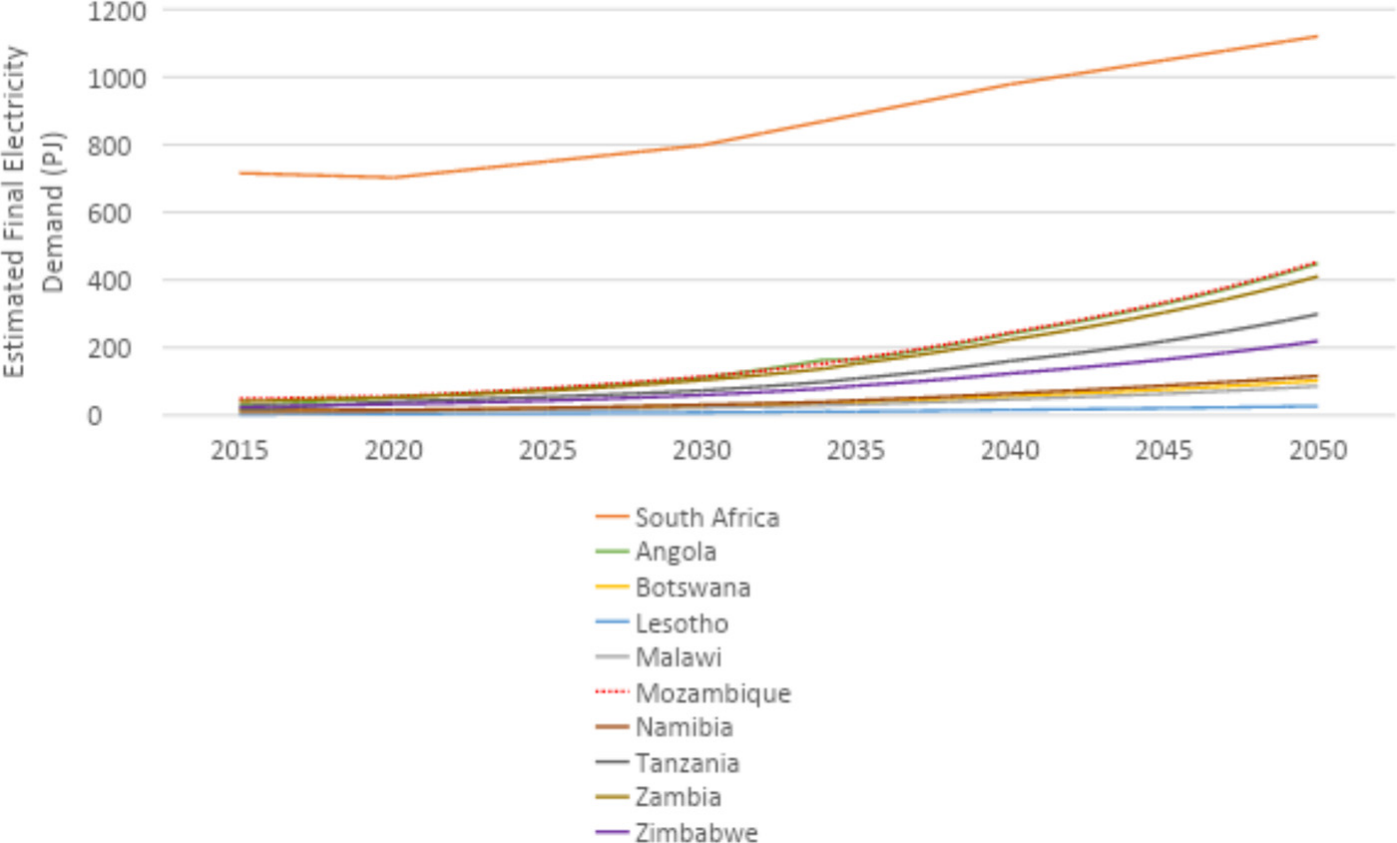
Final electricity demand projection (PJ) for countries in the South Africa Power Pool (source N).

**Fig. 6:**
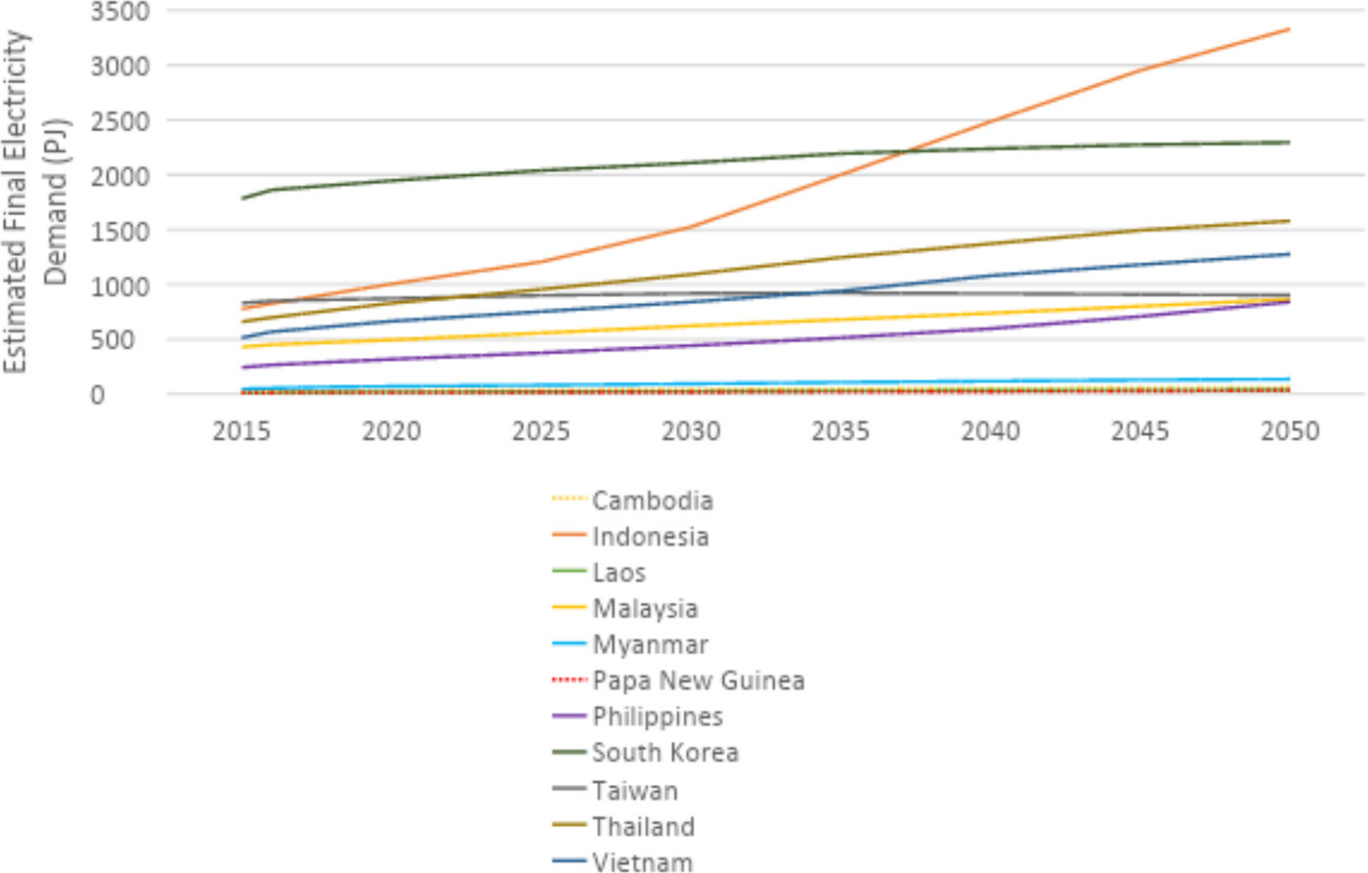
Final electricity demand projection (PJ) for selected countries in East Asia (sources W, AL).

**Fig. 7:**
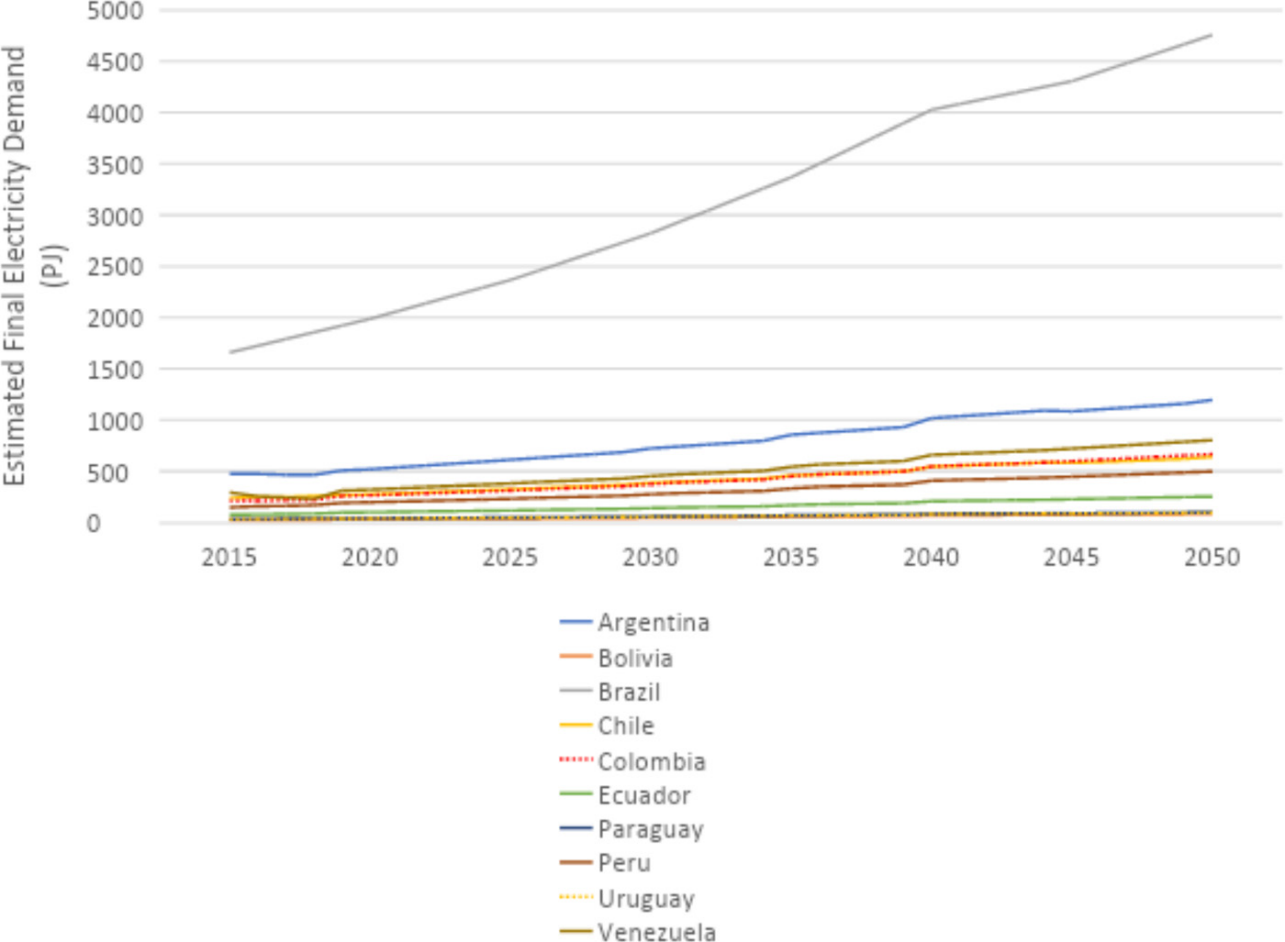
Final electricity demand projection (PJ) for selected countries in South America (sources AL-AM)

Final electricity demand projections from 2015 to 2020 are provided for each country. These projections estimate the future demand for electricity, considering factors such as population growth and industrial activity. For countries in Africa, demand projections were sourced from the reference scenario of the TEMBA study (source N). For countries in Asia, these were sourced from the Business as Usual (BAU) scenario of APEC's 7th Energy Outlook (source W), with growth rates for neighbouring countries and historic consumption (source AL) used to estimate future demand for countries not included in APEC. Demand projections for countries in South America were calculated based on the Current Policy Scenario regional demand projections of the OLADE Energy Outlook 2019 (source AM), which were divided by country based on historical consumption data from the IEA (source AL). For more information on the final electricity demand projection, see Section 2. The figures below show the final electricity demand projections by region for each selected country (Fig. 1, Fig. 2, Fig. 3, Fig. 4, Fig. 5, Fig. 6, Fig. 7).

## 2 Experimental Design, Materials and Methods

Data were primarily collected from the reports and websites of international organizations, including the International Renewable Energy Agency (IRENA), the International Energy Agency (IEA), UN Stats, Asia Pacific Economic Cooperation (APEC), the Economic Research Institute for ASEAN and East Asia (ERIA), Latin America Energy Organisation (OLADE), and the Intergovernmental Panel on Climate Change (IPCC). Additionally, data were sourced from The Electricity Model Base for Africa (TEMBA) and the South America Model Base (SAMBA), existing OSeMOSYS models of African and South American electricity supply (sources K, Q).

### 2.1 Electricity supply system data

Data on the countries’ existing on-grid electricity generation capacity were extracted from the PLEXOS World dataset (sources B-C) using scripts from OSeMOSYS global model generator (source AN). PLEXOS World provides data on the capacity and commissioning date of each power plant. These data were used to estimate installed capacity in future years based on the operational life data in Table 8, Table 9 and 10. Data on the countries’ off-grid renewable energy capacity were sourced from yearly capacity statistics produced by IRENA (source E). Cost, efficiency and operational life data were collected from regional reports by IRENA and ACE and the SAMBA dataset for South America (sources F, G, I, K), which provide region-specific estimates by technology. IRENA's 2021 report focussing on Eastern and Southern Africa (source F) also provides projections of future cost reductions for renewable energy technologies. These future cost projections were used for African countries. At the same time, the trend for each technology was applied to the current regional cost estimates for East Asia and South America to estimate future cost reductions in these regions. For offshore wind, the cost reduction trend was taken from a technology-specific IRENA report on the future of wind (source H) instead since it is not featured in (source F). The resulting projections are presented in Table 11, Table 12 and 13. It was assumed that costs fall linearly between the data points provided by IRENA and that costs remain constant beyond 2040 when the IRENA forecasts end (except for the offshore wind, where the IRENA forecast continues to 2050). Fixed costs for renewable energy technologies in each year were estimated by calculating a certain percentage (ranging from 1 to 4% depending on the technology) of the capital cost in that year, as done by IRENA (source F).

Country-specific capacity factors for solar PV, onshore wind and hydropower in all regions were sourced from Renewables Ninja and the PLEXOS-World 2015 Model Dataset (sources B, C, L, M). These sources provide hourly capacity factors for 2015 for solar PV and wind and 15-year average monthly capacity factors for hydropower. Country-specific capacity factors for offshore wind in Africa were sourced from the TEMBA dataset (sources N, Q), which provides capacity factor estimates for eight timeslices. For countries in East Asia and South America, country-specific capacity factors for offshore wind were estimated based on an NREL source that estimates the potential wind power capacity by capacity factor range in each country (source O), from which a capacity-weighted average was calculated. Average capacity factors are presented in Table 14, Table 15, Table 16 and 16. These data were also used to estimate capacity factors for eight timeslices used in the OSeMOSYS model (see detail in Appendix A). Capacity factors for other technologies were sourced from reports by IRENA for Africa (sources F, G, J), IRENA and ACE for East Asia (source I), and the SAMBA dataset for South America (source K), which provide generic regional estimates for each technology.

The costs and efficiencies of electricity transmission and distribution in Africa were sourced from the TEMBA reference case (source N), which provides generic regional cost estimates and country-specific efficiencies which consider expected efficiency improvements in the future. For East Asia, the combined capital costs of electricity transmission and distribution are estimated based on an ERIA report which gives estimated capital costs for nine projects in ASEAN (source S), with an average value used. The fixed operational cost is assumed to be 2% of the estimated capital cost, as done by ERIA (source S). The combined losses of transmission and distribution in countries in East Asia in 2014 were sourced from IEA data (source R), and it was then assumed that combined losses would fall to 5% by 2050 in a linear fashion from 2014. For countries in South America, the capital costs, operational lives, and efficiencies of electricity transmission and distribution were also taken from the SAMBA dataset (source K), which provides future projections. Techno-economic data for refineries were sourced from the IEA Energy Technology Systems Analysis Programme (ETSAP) (source U), which provides generic estimates of costs and performance parameters. In contrast, the refinery options modelled are based on the methods used in TEMBA (source N). Existing domestic refinery capacities across all regions were sourced from the McKinsey Refinery Reference Desk, which lists refineries by country (source T).

### 2.2 Fuel data

For countries in East Asia, fuel prices for crude oil, diesel, fuel oil, natural gas and coal were taken from the APEC Energy Outlook 7th Edition (source W), which provides cost estimates by fuel from 2016 to 2050. APEC provide different natural gas and coal prices for net importers, exporters, and neutral countries, with the relevant prices used for each country. For countries in Asia, the domestic biomass price was estimated from an ERIA report that gives a local average in Thailand (source X) since this was the most region-specific cost estimate that could be sourced. The imported biomass price is an international average taken from a 2021 biomass markets report by Argus Media (source Y).

For countries in Africa, the crude oil price is based on a global price forecast produced by the US Energy Information Administration (EIA) in 2020, which runs to 2050 (source V). The price was increased by 10% for imported oil to reflect the cost of importation. The imported HFO and LFO costs were calculated by multiplying the oil price by 0.8 and 1.33, respectively, based on the methods used in TEMBA (source Q). The prices of coal, natural gas and biomass in Africa were sourced from a regional IRENA report (source G), which provides generic regional estimates for costs to 2030. Again, a linear rate of change was assumed between data points from IRENA, and the forecast was extended to 2040 using the rate of change between 2020 and 2030. Prices were then assumed constant after 2040. The cost of domestically-produced biomass was increased by 10% to estimate the cost of imported biomass.

For countries in South America, fuel price projections for crude oil were also taken from the same 2020 US EIA international oil price forecast (source V), with the prices for imported HFO and LFO calculated in the same way as for Africa described above. Each country's natural gas price forecast was taken from SAMBA, providing country-specific forecasts for 2063 (source K). The domestic biomass price was estimated based on a UK Government report on international biomass markets (source Z) that includes cost estimates for biomass production in Brazil. This cost was increased by 10% to estimate the price for imported biomass.

### 2.3 Emissions factors and domestic reserves

Emissions factors were collected from the IPCC Emission Factor Database (source AA), which provides carbon emissions factors by fuel.

For countries in Africa, domestic renewable energy potentials for solar PV, Concentrating Solar Power and wind were collected from an IRENA-KTH working paper (source AB), which provides estimates of potential yearly generation by country in Africa. Other renewable energy potentials for countries in Africa were sourced from regional reports by IRENA (sources G, AC, AO) and the World Small Hydropower Development Report (source AD), which provide estimated potentials in MW by country. Estimated domestic fossil fuel reserves for countries in Africa are from the websites of The World Bank and US EIA (sources AH-AI), which provide estimates of reserves by country.

For countries in East Asia, domestic solar PV and onshore wind potentials were primarily collected from an NREL report which provides estimated potential yearly generation with an LCOE under $150/MWh (source AF). For Asian countries not included in that report, the domestic solar and onshore wind resources were collected from other NREL datasets, which provide estimates of potential yearly generation by country (source O, AG). Offshore wind potentials were collected from the wind NREL dataset (source O) where applicable. Other renewable energy potentials in East Asia were sourced from regional reports (source AE, AP) and the World Small Hydropower Development Report (source AD), which provide estimated potentials by country. Estimated domestic fossil fuel reserves were primarily sourced from the APEC Energy Outlook 7th Edition (source W) or Worldometer (source AJ).

Domestic solar and wind resources were also collected from NREL datasets for countries in South America, which provide estimates of potential yearly generation by country (sources O, AG). Other renewable energy potentials were sourced from a regional report by OLADE (source AM) and the World Small Hydropower Development Report (source AD). Estimated domestic coal and oil reserves were sourced from the SAMBA dataset (source K), while natural gas reserves were sourced from the 2019 BP Statistical Review (source AK), which provide estimates of reserves by country.

For the minority of countries not included in one of the regional and global datasets described above, estimates of domestic renewable energy potential and fossil fuel reserves were extracted from country-specific papers and reports. Analysts wishing to use country starter datasets should consult the externally hosted data repository and country-specific preprint article (see Appendix B) to elucidate exactly which source was used for each country.

### 2.4 Electricity demand data

The final electricity demand projections for countries in Africa are based on data from the TEMBA Reference Scenario dataset (source N), which provides yearly total demand estimates from 2015 to 2070 under a reference case scenario. Final electricity demand projections for countries in Asia are collected from the BAU projection from the APEC Energy Outlook 7th Edition (source W), with total demand estimates for every five years from 2015 to 2050, with demand assumed to change linearly between these data points. For Asian countries not included in the APEC Energy Outlook, a demand projection was estimated by applying the trend of the projections for neighbouring countries to the total demand in 2019 from the IEA (source AL). For countries in South America, the final electricity demand projections are based on the Current Policy Scenario of the OLADE Energy Outlook 2019 (source AM), which provides regional aggregated demand projections to 2040. These regional cost projections were divided by country using historical consumption data from the IEA (source AL) and extended to 2070 by extrapolating the growth trend to 2070.

## Ethics Statement

Not applicable.

## Funding

As well as support in kind provided by the employers of the authors of this note, we also acknowledge core funding from the Climate Compatible Growth Program (#CCG) of the UK's Foreign Development and Commonwealth Office (FCDO). The views expressed in this paper do not necessarily reflect the UK government's official policies.

## CRediT authorship contribution statement

**Lucy Allington:** Conceptualization, Data curation, Investigation, Methodology, Writing – original draft, Visualization, Validation. **Carla Cannone:** Conceptualization, Data curation, Investigation, Methodology, Software, Formal analysis, Validation, Visualization, Writing – review & editing. **Ioannis Pappis:** Data curation, Investigation, Validation, Writing – review & editing. **Karla Cervantes Barron:** Data curation, Software, Visualization. **Will Usher:** Software, Supervision. **Steve Pye:** Supervision, Project administration. **Edward Brown:** . **Mark Howells:** Conceptualization, Methodology, Writing – review & editing, Supervision. **Miriam Zachau Walker:** Investigation, Software. **Aniq Ahsan:** Investigation, Software. **Flora Charbonnier:** Investigation, Software. **Claire Halloran:** Investigation, Software. **Stephanie Hirmer:** Investigation, Supervision, Writing – review & editing. **Jennifer Cronin:** Writing – review & editing. **Constantinos Taliotis:** Conceptualization, Writing – review & editing. **Caroline Sundin:** Conceptualization, Writing – review & editing. **Vignesh Sridharan:** Conceptualization. **Eunice Ramos:** Conceptualization. **Maarten Brinkerink:** Data curation. **Paul Deane:** Data curation. **Andrii Gritsevskyi:** Conceptualization. **Gustavo Moura:** Data curation. **Arnaud Rouget:** Conceptualization. **David Wogan:** Conceptualization. **Edito Barcelona:** Conceptualization. **Taco Niet:** Writing – review & editing. **Holger Rogner:** Conceptualization. **Franziska Bock:** Writing – review & editing. **Jairo Quirós-Tortós:** Validation, Writing – review & editing, Data curation. **Jam Angulo-Paniagua:** Validation, Writing – review & editing. **Satheesh Krishnamurthy:** Writing – review & editing. **John Harrison:** Writing – review & editing. **Long Seng To:** Writing – review & editing.

## Declaration of Competing Interest

The authors declare that they have no known competing financial interests or personal relationships which have or could be perceived to have influenced the work reported in this article.

## Figures and Tables

**Table A1:** Definitions of the three model scenarios.

Scenario	Definition
*Fossil Future*	No new investments in renewable or nuclear power generation, electric stoves and heating, electric transport or energy efficiency are permitted.
*Least Cost*	No new investment in nuclear power is permitted. Gradual investment constraints are applied to demand-side fuel-switching and energy efficiency. Only up to 5% of each technology's 2050 capacity run without demand-side investment constraints can be invested annually. No additional constraints are applied to find the cost-optimal solution.
*Net Zero by 2050*	Domestic production and imports of fossil fuels and biomass gradually decline to 0 in 2050, beginning in 2021, leading to zero carbon emissions by 2050. No new investment in nuclear power is permitted. Gradual investment constraints are applied to demand-side fuel-switching and energy efficiency. Only up to 5% of each technology's 2050 capacity in a run without demand-side investment constraints can be invested annually from 2021 to 2039, rising to 10% from 2040 to 2050 to reflect greater ambition.

**Fig. A1:**
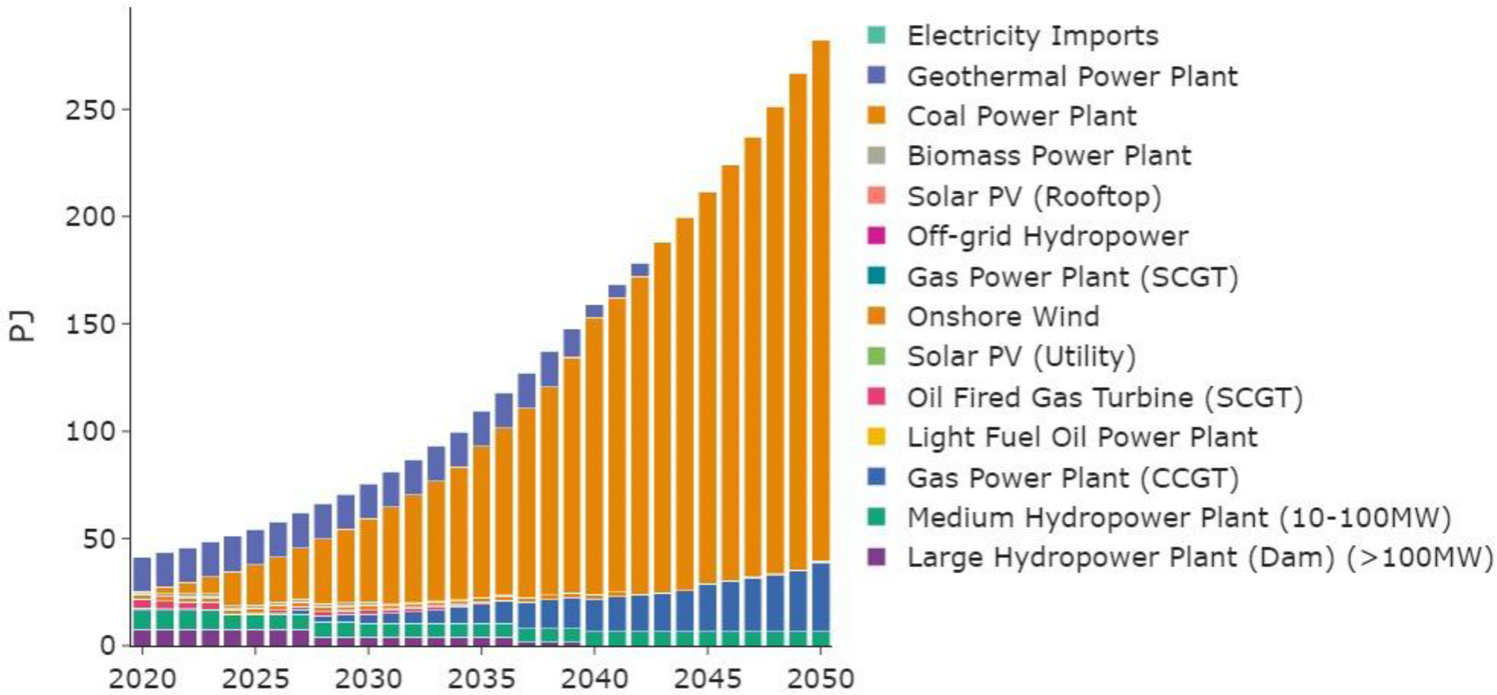
Electricity generation by technology type in Kenya in the Fossil Future scenario.

**Fig. A2:**
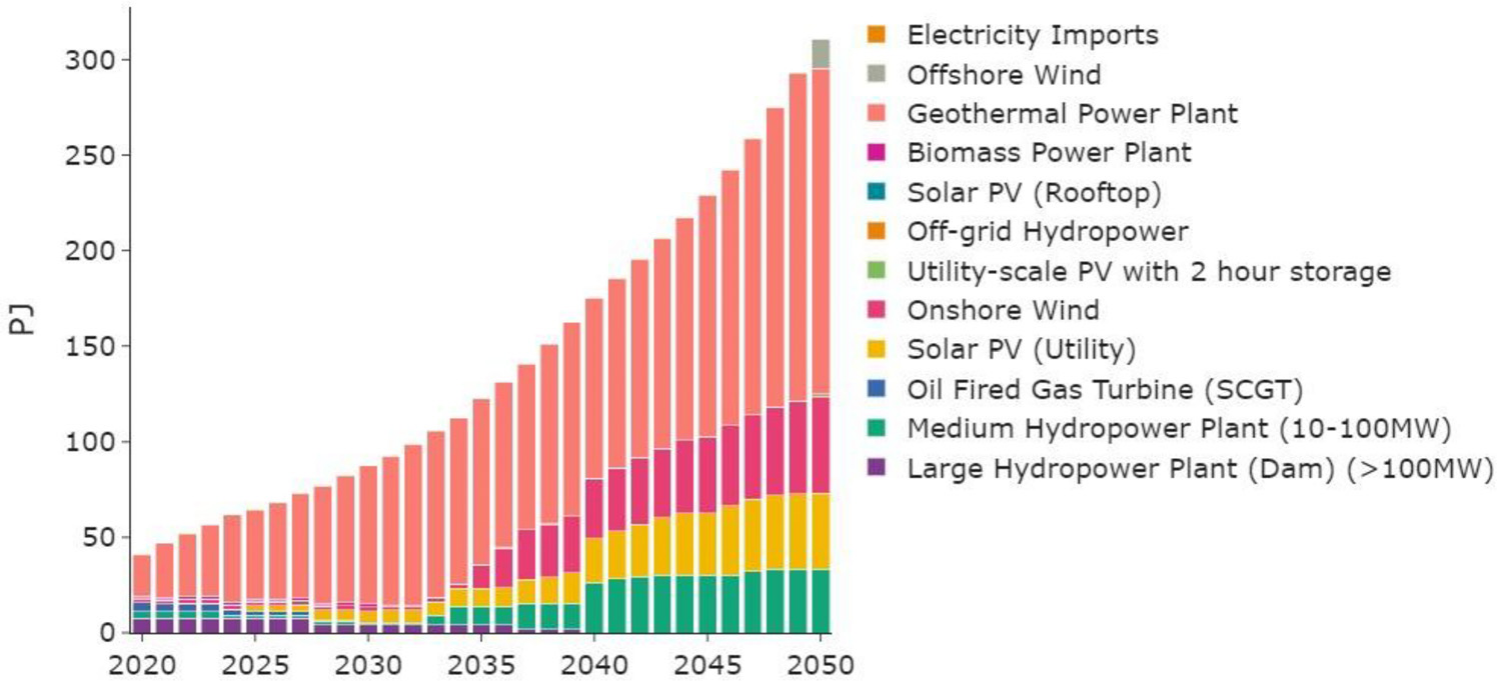
Electricity generation by technology type in Kenya in the Least Cost scenario.

**Fig. A3:**
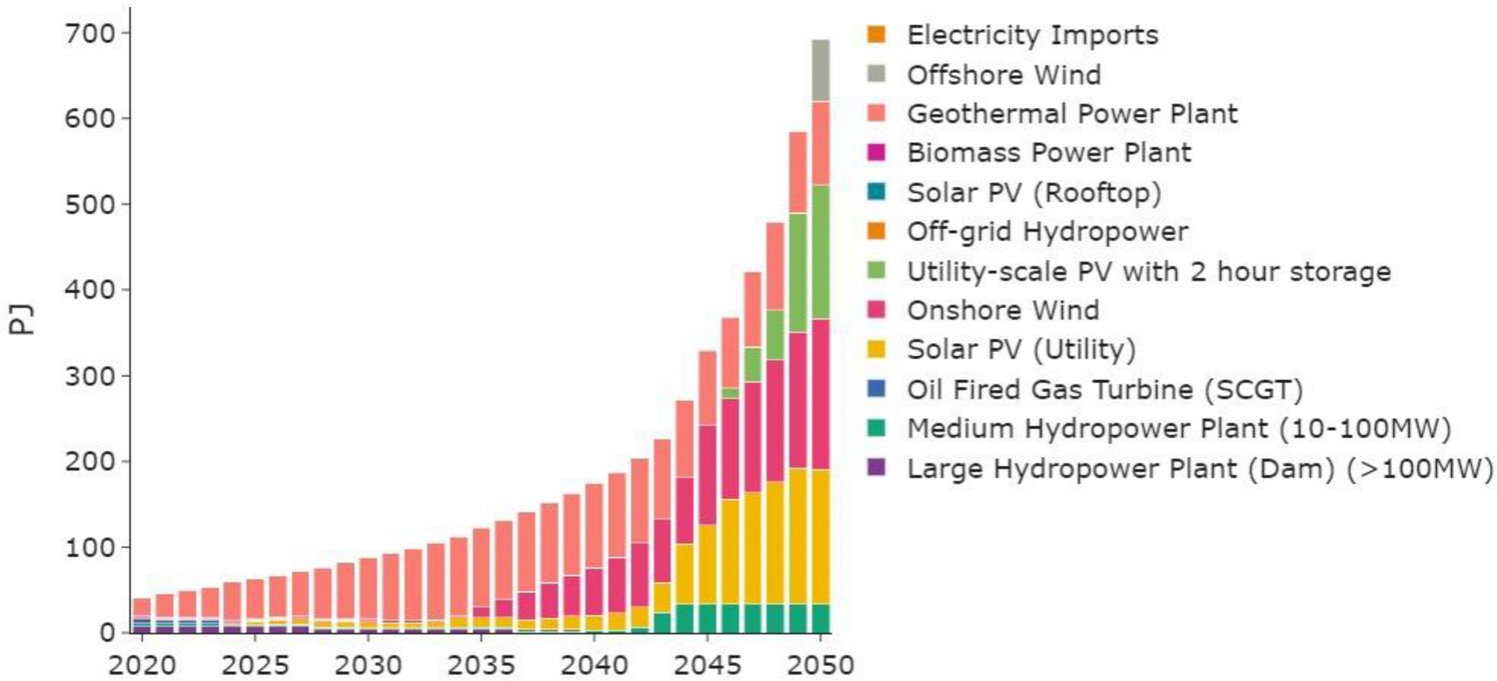
Electricity generation in Kenya by technology type in the Net Zero by 2050 scenario.

**Fig. A4:**
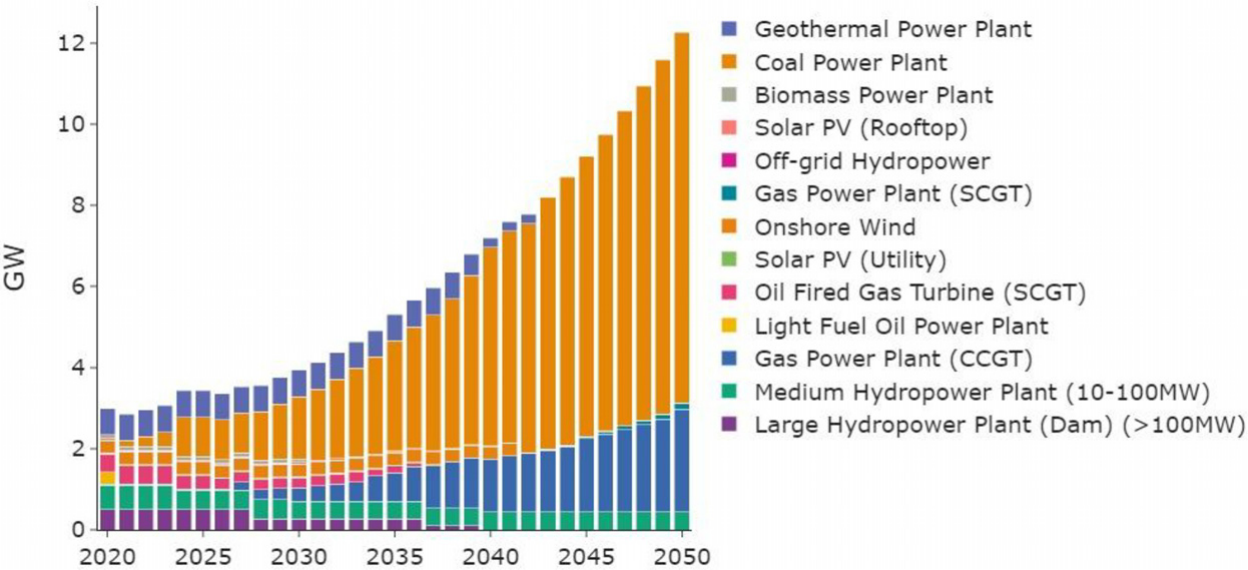
Installed capacity by technology type in Kenya in the Fossil Future scenario.

**Fig. A5:**
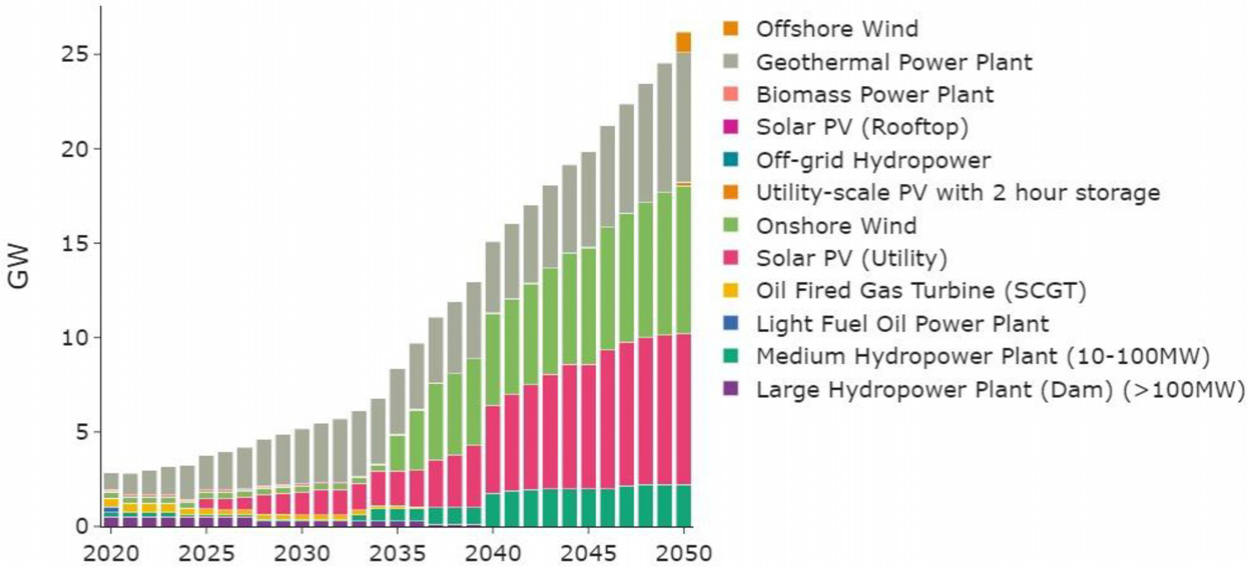
Installed capacity by technology type in Kenya in the Least Cost scenario.

**Fig. A6:**
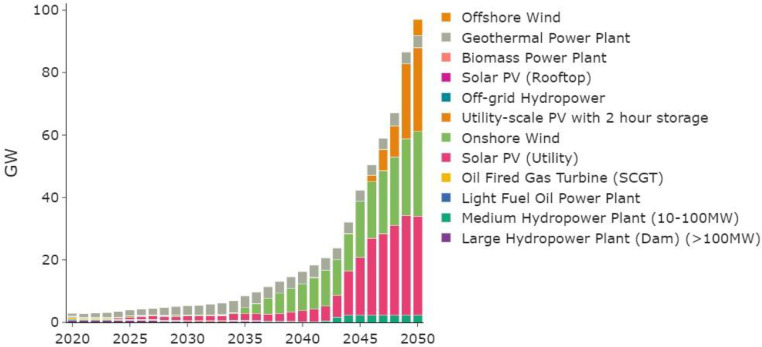
Installed capacity by technology type in Kenya in the Net Zero scenario.

**Fig. A7:**
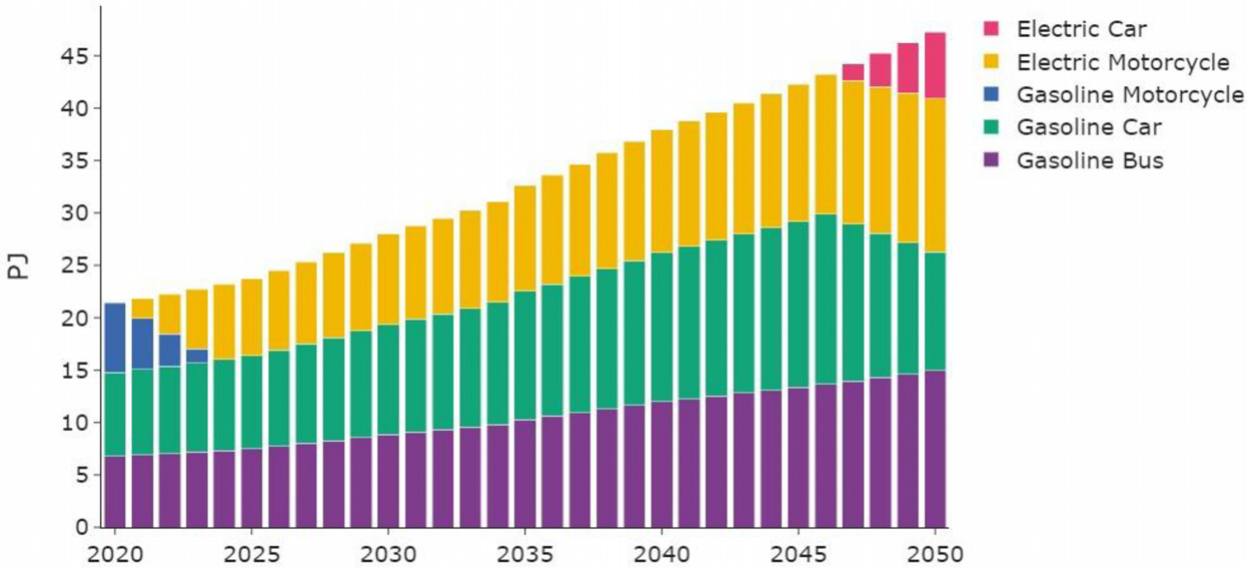
Transport demand met by each technology in Kenya in the Least Cost scenario.

**Fig. A8:**
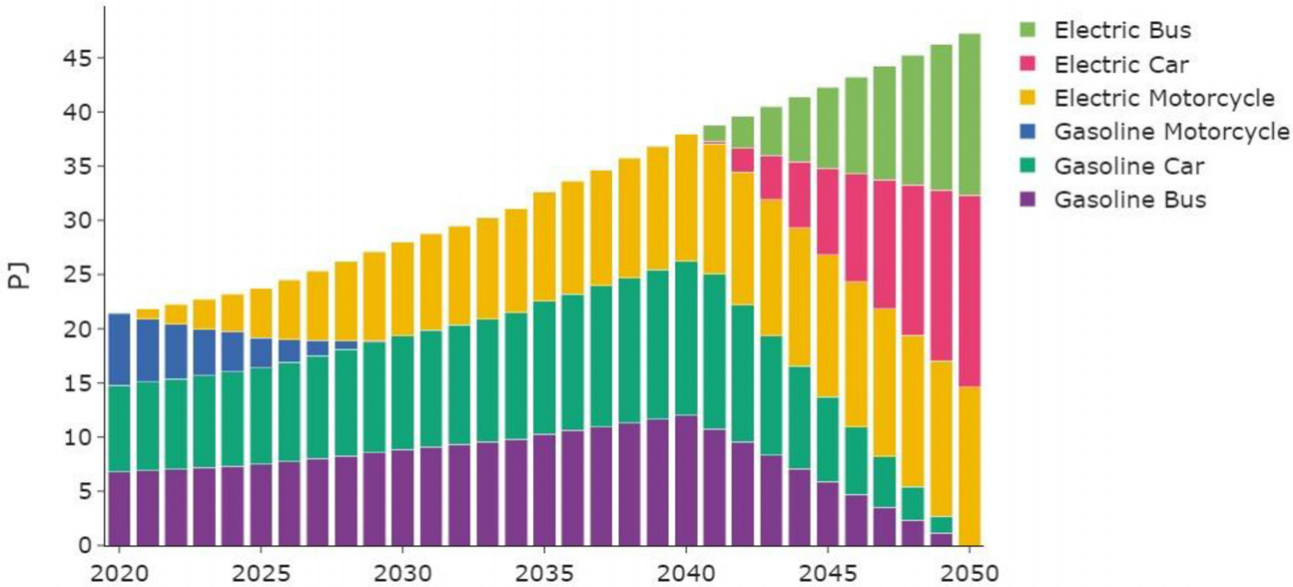
Transport demand met by each technology in Kenya in the Net Zero by 2050 scenario.

**Fig. A9:**
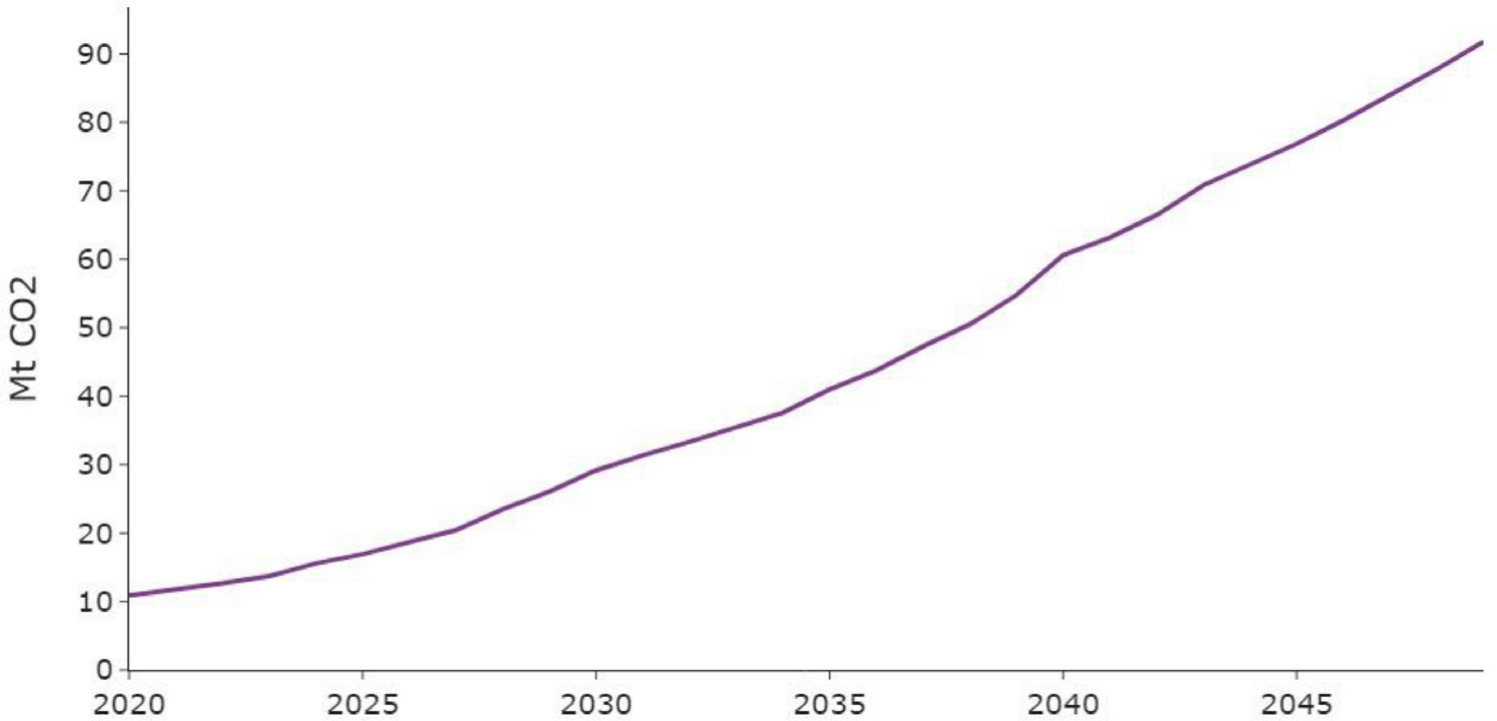
Annual carbon dioxide emissions from electricity generation in Kenya in the Fossil Future scenario.

**Fig. A10:**
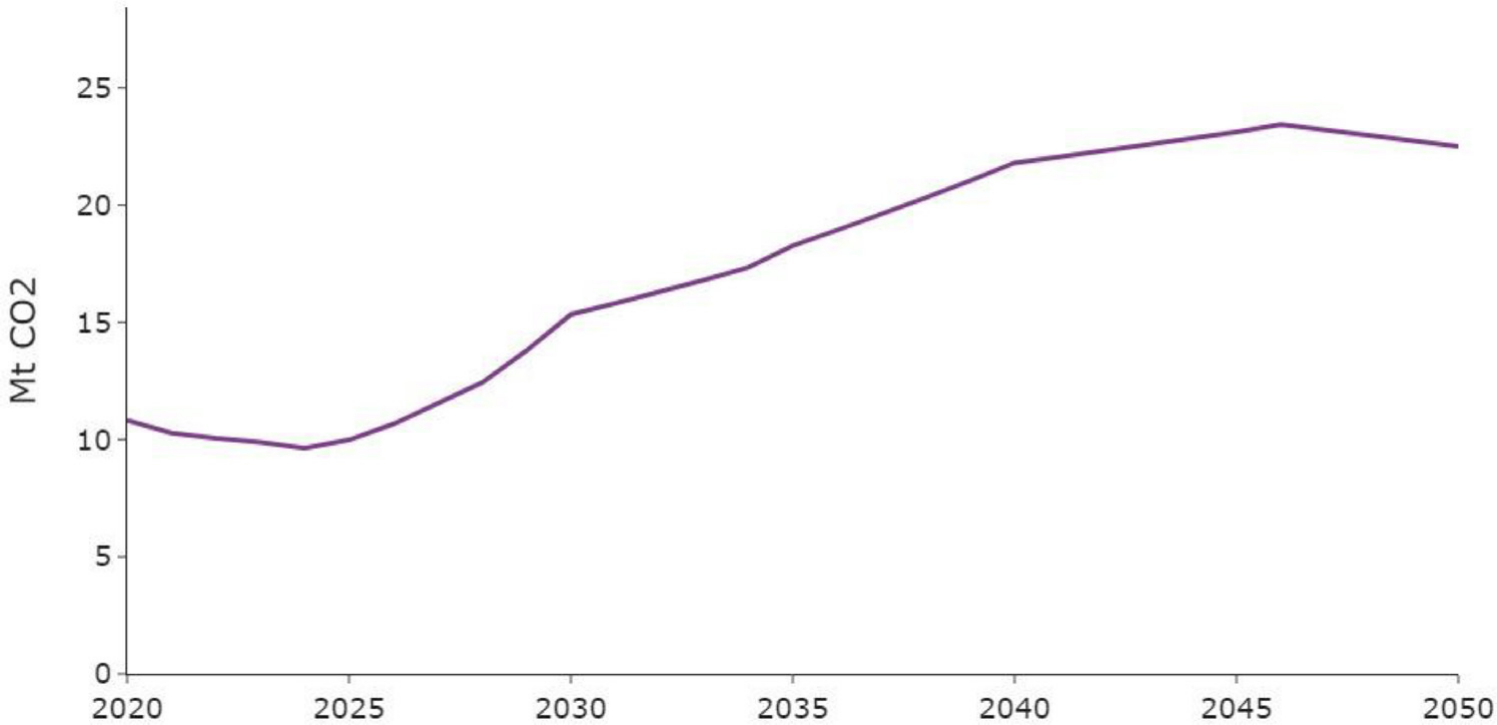
Annual carbon dioxide emissions from electricity generation in Kenya in the Least Cost scenario.

**Fig. A11:**
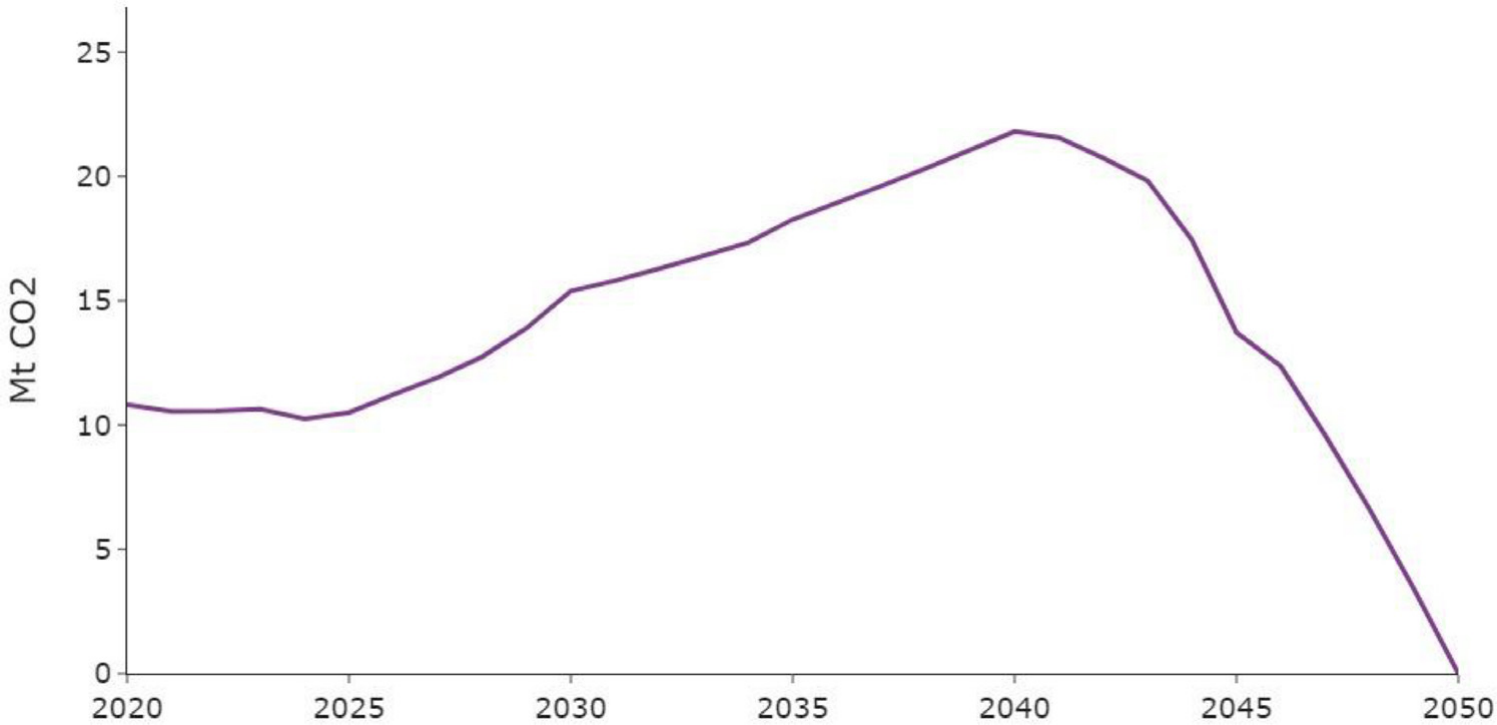
Annual carbon dioxide emissions from electricity generation in Kenya in the Net Zero scenario.

**Fig. A1:**
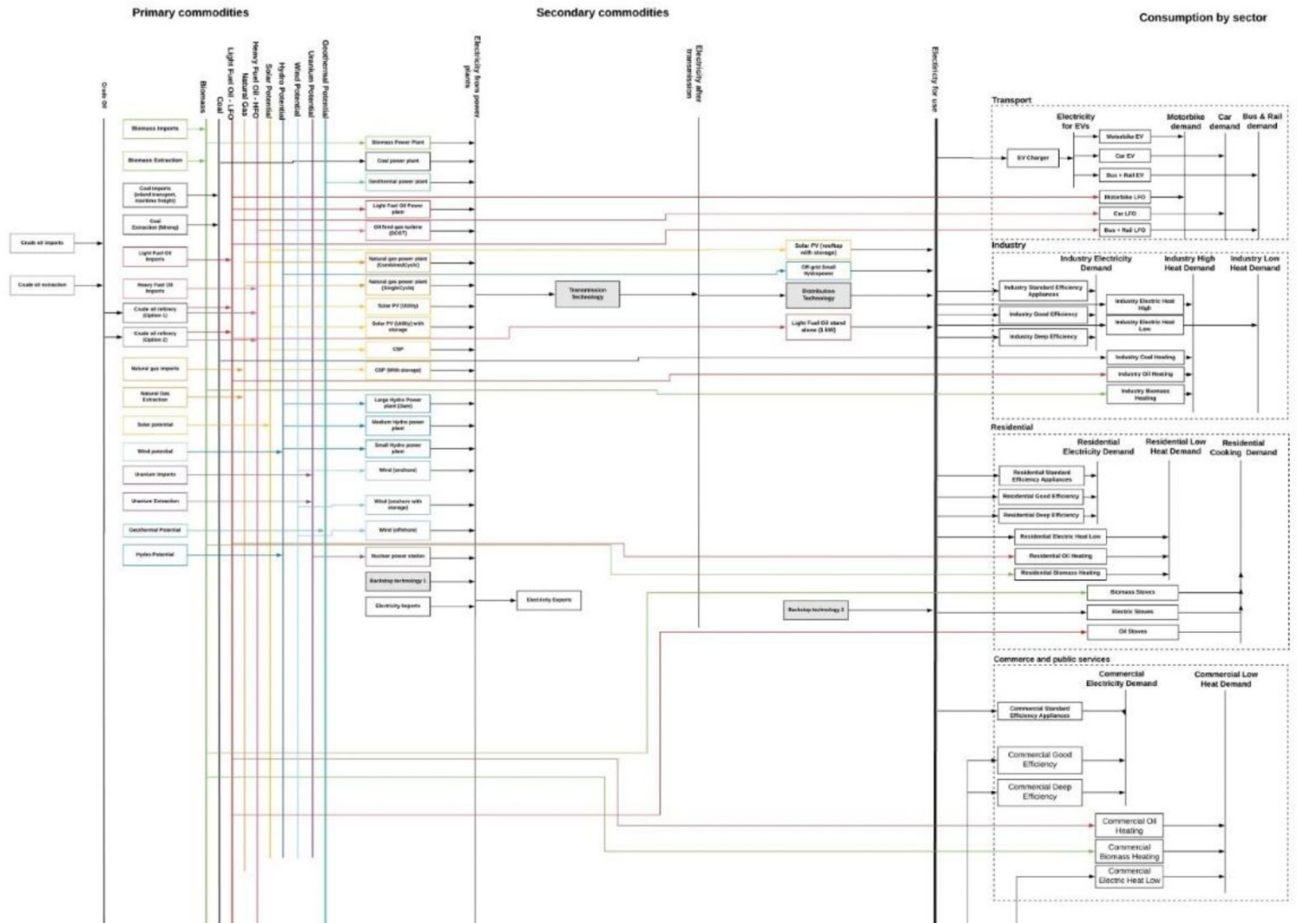
Reference Energy System

**Table B1:** External country datasets created using the data described in this article.

Region	Country	Zenodo Dataset	ResearchSquare Pre-Print
Africa	Algeria	https://doi.org/10.5281/zenodo.4728143	https://www.researchsquare.com/article/rs-478421/v2
Africa	Angola	https://doi.org/10.5281/zenodo.4650810	https://www.researchsquare.com/article/rs-478581/v2
Africa	Benin	https://doi.org/10.5281/zenodo.4725486	https://www.researchsquare.com/article/rs-478594/v2
Africa	Botswana	https://doi.org/10.5281/zenodo.4650986	https://www.researchsquare.com/article/rs-478620/v2
Africa	Burkina Faso	https://doi.org/10.5281/zenodo.4650942	https://www.researchsquare.com/article/rs-478764/v2
Africa	Burundi	https://doi.org/10.5281/zenodo.4725445	https://www.researchsquare.com/article/rs-478806/v2
Africa	Cameroon	https://doi.org/10.5281/zenodo.4650822	https://www.researchsquare.com/article/rs-478850/v2
Africa	Central African Republic	https://doi.org/10.5281/zenodo.4650968	https://www.researchsquare.com/article/rs-478906/v2
Africa	Chad	https://doi.org/10.5281/zenodo.4725466	https://www.researchsquare.com/article/rs-478927/v2
Africa	Côte d'Ivoire	https://doi.org/10.5281/zenodo.4737634	https://www.researchsquare.com/article/rs-493226/v1
Africa	Democratic Republic of the Congo	https://doi.org/10.5281/zenodo.4737640	https://www.researchsquare.com/article/rs-493235/v1
Africa	Djibouti	https://doi.org/10.5281/zenodo.4725462	https://www.researchsquare.com/article/rs-479210/v2
Africa	Egypt	https://doi.org/10.5281/zenodo.4652804	https://www.researchsquare.com/article/rs-479263/v2
Africa	Equatorial Guinea	https://doi.org/10.5281/zenodo.4650904	https://www.researchsquare.com/article/rs-479310/v2
Africa	Eritrea	https://doi.org/10.5281/zenodo.4725456	https://www.researchsquare.com/article/rs-479568/v2
Africa	Eswatini (fmr. "Swaziland")	https://doi.org/10.5281/zenodo.4737638	https://www.researchsquare.com/article/rs-493243/v1
Africa	Ethiopia	https://doi.org/10.5281/zenodo.4650876	https://www.researchsquare.com/article/rs-479603/v2
Africa	Gabon	https://doi.org/10.5281/zenodo.4737642	https://www.researchsquare.com/article/rs-493249/v1
Africa	Gambia	https://doi.org/10.5281/zenodo.4651140	https://www.researchsquare.com/article/rs-479641/v2
Africa	Ghana	https://doi.org/10.5281/zenodo.4725480	https://www.researchsquare.com/article/rs-479778/v2
Africa	Guinea	https://doi.org/10.5281/zenodo.4725454	https://www.researchsquare.com/article/rs-480013/v3
Africa	Guinea-Bissau	https://doi.org/10.5281/zenodo.4650850	https://www.researchsquare.com/article/rs-480393/v1
Africa	Kenya	https://doi.org/10.5281/zenodo.4650874	https://www.researchsquare.com/article/rs-480458/v1
Africa	Lesotho	https://doi.org/10.5281/zenodo.4650866	https://www.researchsquare.com/article/rs-480748/v1
Africa	Liberia	https://doi.org/10.5281/zenodo.4650794	https://www.researchsquare.com/article/rs-480654/v1
Africa	Libya	https://doi.org/10.5281/zenodo.4650920	https://www.researchsquare.com/article/rs-481132/v1
Africa	Malawi	https://doi.org/10.5281/zenodo.4652798	https://www.researchsquare.com/article/rs-479507/v2
Africa	Mali	https://doi.org/10.5281/zenodo.4725447	https://www.researchsquare.com/article/rs-479627/v2
Africa	Mauritania	https://doi.org/10.5281/zenodo.4650914	https://www.researchsquare.com/article/rs-479591/v2
Africa	Morocco	https://doi.org/10.5281/zenodo.4725482	https://www.researchsquare.com/article/rs-480023/v2
Africa	Mozambique	https://doi.org/10.5281/zenodo.4650902	https://www.researchsquare.com/article/rs-481070/v1
Africa	Namibia	https://doi.org/10.5281/zenodo.4652808	https://www.researchsquare.com/article/rs-481002/v1
Africa	Niger	https://doi.org/10.5281/zenodo.4725476	https://www.researchsquare.com/article/rs-480051/v2
Africa	Nigeria	https://doi.org/10.5281/zenodo.4728145	https://www.researchsquare.com/article/rs-480085/v2
Africa	Congo (Congo-Brazzaville)	https://doi.org/10.5281/zenodo.4651133	https://www.researchsquare.com/article/rs-479154/v2
Africa	Rwanda	https://doi.org/10.5281/zenodo.4652800	https://www.researchsquare.com/article/rs-480847/v1
Africa	Senegal	https://doi.org/10.5281/zenodo.4725484	https://www.researchsquare.com/article/rs-480122/v2
Africa	Sierra Leone	https://doi.org/10.5281/zenodo.4725544	https://www.researchsquare.com/article/rs-480371/v2
Africa	Somalia	https://doi.org/10.5281/zenodo.4725474	https://www.researchsquare.com/article/rs-480695/v1
Africa	South Africa	https://doi.org/10.5281/zenodo.4652802	https://www.researchsquare.com/article/rs-480636/v1
Africa	South Sudan	https://doi.org/10.5281/zenodo.4725468	https://www.researchsquare.com/article/rs-479969/v2
Africa	Sudan	https://doi.org/10.5281/zenodo.4725460	https://www.researchsquare.com/article/rs-479952/v2
Africa	Tanzania	https://doi.org/10.5281/zenodo.4652806	https://www.researchsquare.com/article/rs-481182/v1
Africa	Togo	https://doi.org/10.5281/zenodo.4725451	https://www.researchsquare.com/article/rs-480160/v2
Africa	Tunisia	https://doi.org/10.5281/zenodo.4725458	https://www.researchsquare.com/article/rs-480566/v1
Africa	Uganda	https://doi.org/10.5281/zenodo.4652795	https://www.researchsquare.com/article/rs-480512/v1
Africa	Zambia	https://doi.org/10.5281/zenodo.4725470	https://www.researchsquare.com/article/rs-480042/v2
Africa	Zimbabwe	https://doi.org/10.5281/zenodo.4650816	https://www.researchsquare.com/article/rs-479655/v2
Asia	Cambodia	https://doi.org/10.5281/zenodo.5139538	https://www.researchsquare.com/article/rs-757472/v1
Asia	Indonesia	https://doi.org/10.5281/zenodo.4926858	https://www.researchsquare.com/article/rs-757493/v1
Asia	Laos	https://doi.org/10.5281/zenodo.4926880	https://www.researchsquare.com/article/rs-757542/v1
Asia	Malaysia	https://doi.org/10.5281/zenodo.5139480	https://www.researchsquare.com/article/rs-757581/v1
Asia	Myanmar (formerly Burma)	https://doi.org/10.5281/zenodo.5139484	https://www.researchsquare.com/article/rs-757622/v1
Asia	Philippines	https://doi.org/10.5281/zenodo.5139542	https://www.researchsquare.com/article/rs-757671/v1
Asia	South Korea	https://doi.org/10.5281/zenodo.5139512	https://www.researchsquare.com/article/rs-757722/v1
Asia	Taiwan	https://doi.org/10.5281/zenodo.5139520	https://www.researchsquare.com/article/rs-757733/v1
Asia	Thailand	https://doi.org/10.5281/zenodo.5139498	https://www.researchsquare.com/article/rs-757735/v1
Asia	Vietnam	https://doi.org/10.5281/zenodo.5139527	https://www.researchsquare.com/article/rs-757746/v1
Asia	Papua New Guinea	https://doi.org/10.5281/zenodo.5139488	https://www.researchsquare.com/article/rs-757653/v1
Latin America	Argentina	https://doi.org/10.5281/zenodo.5498081	https://www.researchsquare.com/article/rs-893102/v1
Latin America	Bolivia	https://doi.org/10.5281/zenodo.5498083	https://www.researchsquare.com/article/rs-893267/v1
Latin America	Brazil	https://doi.org/10.5281/zenodo.5498085	https://www.researchsquare.com/article/rs-893535/v1
Latin America	Chile	https://doi.org/10.5281/zenodo.5498087	https://www.researchsquare.com/article/rs-893607/v1
Latin America	Colombia	https://doi.org/10.5281/zenodo.5498091	https://www.researchsquare.com/article/rs-893706/v1
Latin America	Ecuador	https://doi.org/10.5281/zenodo.5498093	https://www.researchsquare.com/article/rs-893779/v1
Latin America	Paraguay	https://doi.org/10.5281/zenodo.5498099	https://www.researchsquare.com/article/rs-895567/v1
Latin America	Peru	https://doi.org/10.5281/zenodo.5498101	https://www.researchsquare.com/article/rs-895579/v1
Latin America	Uruguay	https://doi.org/10.5281/zenodo.5498103	https://www.researchsquare.com/article/rs-895585/v1
Latin America	Venezuela	https://doi.org/10.5281/zenodo.5498105	https://www.researchsquare.com/article/rs-895585/v1

## Data Availability

Starter Kit Data (Reference data) (Zenodo).

## Appendix A – Zero-order tier 2 OSeMOSYS model

The data described above were used to create a simple zero-order Tier 2 energy systems model for Kenya. As it is open-source and free, an OSeMOSYS model is calibrated and run with three example scenarios. Note that these scenarios in no way represent the development trajectories of the country. This model and its results are intended to act as an example of what can be produced using the data in this article and a starting point for further model development.

In the OSeMOSYS model, the electricity supply system is represented by importing and extraction technologies, conversion technologies, power plants, transmission and distribution network systems and final energy demands for the different available fuels considered. This is shown diagrammatically in the Reference Energy System shown below. The main modeling assumptions consist of electricity generation capacity per type of technology (on- and off-grid), fuel prices, emissions, transmission and distribution network capacity and losses, and refineries, which are exogenous parameters in the model. Furthermore, the final exogenous energy demands entered into the model are disaggregated by fuel and sector. The data described in this article were used as input data to define these assumptions in the model.

### A1 Model assumptions

Key assumptions used in model development are outlined below.

**Supply-side assumptions**

The share of total demand that can be met by off-grid solar PV is constrained based on the optimal balance of on- and off-grid provision in a least-cost scenario for 100% electricity access by 2030 from the Global Electrification Platform (source AQ). Other technologies were modelled to represent utility-scale solar PV and onshore wind with storage capacity. Utility-scale PV with two-hour storage and onshore wind with half-hour storage were modelled, with the additional costs of storage estimated based on data from the NREL ATB 2020 Database, which provides cost projections for different durations of storage up to 2050 (source AR). The maximum share of total demand that variable renewables can meet is constrained as follows: utility-scale PV, onshore wind and utility-scale PV with storage are each permitted to meet up to 15% of demand; offshore wind can meet up to 10% of demand and onshore wind with storage can meet up to 25% of demand. This analysis is not intended to offer a detailed study of system flexibility; however, these constraints ensure the system operates under high renewable shares. Biomass is permitted to meet up to 30% of electricity demand. Electricity imports and exports were modelled simplified, whereby single import and export technologies are constrained to import and export electricity in line with energy balance data (source AL).

**Demand-side assumptions**

Generic techno-economic data for demand-side technologies (cooking, heating and transport) were used (sources AS-AT). The total final electricity demand projection was split by sector based on the proportions of demand in historical energy balance data (source AL). In each sector, moderate and high energy efficiency technologies were modelled, with input activity ratios of 1 and output activity ratios of 1.15 and 1.3, respectively. This is a simplified way of allowing the model to invest in energy efficiency in each sector, with costs estimated based on electricity generation costs by a coal power plant in the model. In the Least Cost and Net Zero scenario (detailed in Section A2), there is a constraint on the speed at which fuel switching and energy efficiency investments can occur to align results to reality better. This is done by limiting the annual investment in electric vehicles, stoves, heating technologies and energy efficiency to 5% of the 2050 capacity.

**Time representation and discount rate**

Within each model year, four seasons, each with two 12 h dayparts, are defined. Daypart 1 starts at 06:00 and finishes at 18:00, while daypart 2 starts at 18:00 and finishes at 06:00. The seasons are defined so that season 1 runs from December to February, season 2 runs from March to May, season 3 from June to August, and season 4 from September to November. A discount rate of 10% is used.

### A2 Scenario definitions

Three stylized scenarios are modelled: Fossil Future, Least Cost and Net Zero by 2050. These scenarios are defined in the table below. Nuclear power is not considered in any of these scenarios; however, it can be added using the techno-economic data provided in the main article.

### A3 Scenario results for Kenya

The graphs below show selected results for the three modelled scenarios, including yearly electricity generation and supply capacity, fuel use in the transport sector and total annual carbon dioxide emissions for 2020–2050.

#### A3.1 Electricity generation results

Fig. A1, Fig. A2–A3

#### A3.2 Capacity expansion results

Fig. A4, Fig. A5–A6

#### A3.3 Transport results

Figs. A7, A8

#### A3.4 Annual carbon dioxide emissions results

Fig. A9, Fig. A10–A11

### A4 further work

These example results represented zero-order models and were generated using the clicSAND Interface [8] and OSeMOSYS code [6]. Those interested in further developing this work are directed to external comprehensive country datasets (see Appendix B) and guidance on model development using clicSAND and OSeMOSYS [9].

## Appendix B – country datasets

Table B1 lists the country-specific datasets that have been created using the data described in this article. For each country, there is a Zenodo dataset which includes the data in a set of csv tables, and a Research Square pre-print article that describes the data collection process and provides stylised example scenarios created using OSeMOSYS. These can act as the basis for country-level analyses such as those on Morocco [3], Ghana [4], and Kenya [5].
